# Multicomponent Oxicam–Metformin Salts: Toward a Strategy for Enhancing Solubility and Stability

**DOI:** 10.1021/acs.cgd.5c01574

**Published:** 2026-02-21

**Authors:** Estephany Muñoz-Hernández, Carolina Alarcón-Payer, Antonio Frontera, Antonio Rodríguez-Diéguez, Francisco J. Acebedo-Martínez, Alicia Domínguez-Martín, Duane Choquesillo-Lazarte

**Affiliations:** † Laboratorio de Estudios Cristalográficos, 16379 IACT-CSIC, Avda. de las Palmeras 4, 18100 Armilla, Spain; ‡ Department of Inorganic Chemistry, Faculty of Pharmacy, 16741 University of Granada, 18071 Granada, Spain; § Servicio de Farmacia, Hospital Universitario Virgen de las Nieves, 18014 Granada, Spain; ∥ Departament de Química, 16745 Universitat de les Illes Balears, Crta. de Valldemossa km 7.5, 07122 Palma, Spain; ⊥ Department of Inorganic Chemistry, Faculty of Sciences, University of Granada, 18071 Granada, Spain

## Abstract

Drug–drug pharmaceutical multicomponent materials (PMMs) offer a promising strategy to modulate the physicochemical properties of active pharmaceutical ingredients, while enabling synergistic effects and combination therapy. Here, we report the preparation and full characterization of a new family of oxicam–metformin (**MTF**) salts, involving the nonsteroidal anti-inflammatory drugs piroxicam (**PRX**), meloxicam (**MLX**), and tenoxicam (**TNX**). Structural and computational studies revealed the role of supramolecular synthons in directing the salt formation and highlighted the relationship between molecular packing and physicochemical properties. Stability analyses showed that these materials enhance **MTF** stability, while particularly protecting **PRX** from hydration. Importantly, incorporation of **MTF** increased the aqueous solubility of the oxicams, while salt formation moderated the excessive solubility of free **MTF**. Significant modifications in fluorescence behavior were also observed, arising from interactions between functional groups involved in the fluorescence procedure within the frameworks. Overall, this study broadens the structural and functional landscape of oxicam–MTF salts and provides a rational framework for designing solid forms with improved stability and solubility.

## Graphical abstract

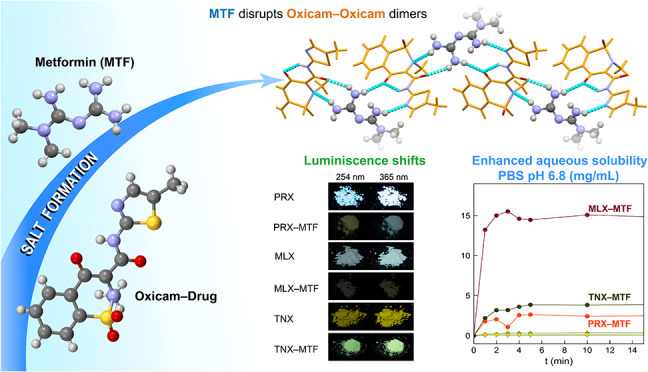

## 1 Introduction

Approximately 85% of pharmaceutical drugs produced in the United States and Europe are administered orally.^1–3^ However, nearly 40% of marketed drugs exhibit poor bioavailability and therapeutic efficacy due to their low aqueous solubility.^4–6^ Hence, improving the solubility of active pharmaceutical ingredients (APIs) has become a primary focus for the pharmaceutical industry.^4–7^

Among the various strategies to optimize drug solubility, the design of pharmaceutical multicomponent materials (PMMs) has emerged as a promising approach due to its successful outcomes.^8^ These systems consist of crystalline solids in which two or more molecular components, at least one of which is an API, cocrystallize in a defined stoichiometric ratio. The resulting crystal structure is stabilized through noncovalent interactions, thereby preserving the molecular integrity and pharmacological activity of the API while enabling modulation of its physicochemical properties.^9–11^ This methodology has been widely applied to poorly soluble APIs, especially those classified by the Biopharmaceutics Classification System (BCS) as class II and IV drugs. However, the same principles can be extended to APIs that have excessively high solubility, which can also present formulation and size effects challenges.^12,13^

Metformin (**MTF**) (Figure 1) is an oral antihyperglycemic agent commonly used in the treatment of type 2 diabetes. Commercialized as a hydrochloride salt (**MTF·HCl**), it is classified as a BCS class III drug, exhibiting low permeability but high solubility.^14,15^ This pharmacokinetic profile contributes to low intestinal absorption, particularly in chronic use, leading to salt accumulation and associated adverse effects such as gastrointestinal discomfort and disorders.^16–18^

**Figure 1:**
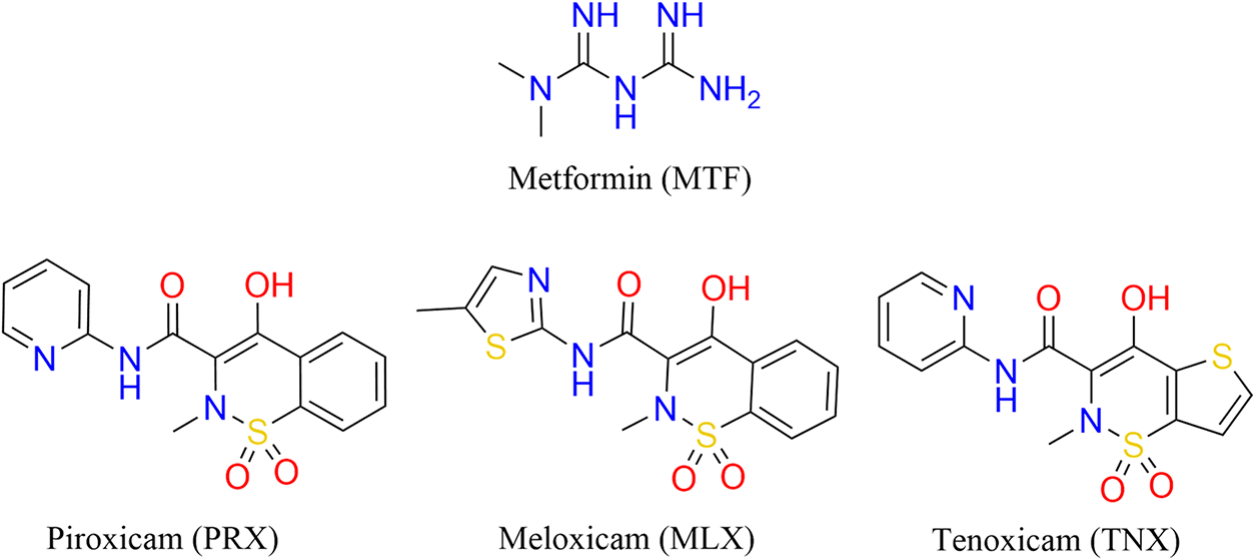
Chemical structures of **MTF** and three selected oxicams used in this work.

Previous studies have shown that forming PMMs with appropriate coformers can successfully tune the properties of **MTF**. Examples include coformers such as saccharin, epalrestat, carboxylic acids and rosiglitazone, among others^19–23^ These combinations not only modulate the solubility of **MTF** but also, when the coformer is a second API, enable the development of drug–drug PPMs with dual, and even potential synergistic therapeutic effects.^24–28^

This strategy is particularly relevant in the context of diabetes, where comorbidities such as arthritis, rheumatism, and peripheral neuropathic pain are prevalent.^29–33^ Diabetic neuropathy, for example, can result in chronic pain, sensory loss, and increased risk of complications such as foot ulcers and infections.^34,35^ These conditions are often managed with nonsteroidal anti-inflammatory drugs (NSAIDs), making their coadministration with **MTF** a common clinical practice.^36–39^

Within the NSAID class, the oxicam family is of particular interest for PMM design. These compounds are BCS class II drugs with intrinsically poor aqueous solubility, making solubility enhancement a key challenge.^40^ Moreover, their structural features, including tautomerism, polymorphism, and in some cases a propensity to form hydrates, confer a high degree of crystalline plasticity, which favors reactivity and salt formation.^41–43^ Although oxicams are not the most common NSAIDs administered with **MTF** in clinical settings, their combination with **MTF** offers an attractive model to explore novel salt systems with improved pharmaceutical performance.

In this study, we explore the development of three dual–drug PMMs combining **MTF** with different NSAIDs, specifically from the Oxicam family belonging to the BCS class II: piroxicam (**PRX**), meloxicam (**MLX**), and tenoxicam (**TNX**) (Figure 1). Our approach aims to simultaneously reduce the solubility of **MTF**, enhance the solubility of the oxicam drugs, and finally set the base for future studies about the possible synergistic therapeutic effects of the novel materials.

## 2 Experimental Section

### 2.1 Materials

All APIs and solvents used in this study were purchased from commercial suppliers. **PRX**, **MLX**, and **TNX** were obtained from TCI Europe (Zwijndrecht, Belgium), and **MTF·HCl** was purchased from Fagron Iberica S.A.U. The solvents Ethyl Acetate (ETA), dichloromethane (DCM), ethanol (EOH) and acetone (ACE) were obtained from Labkem (Barcelona, Spain). Methanol (MET) was sourced from Scharlau S.L. (Spain), while Isopropanol (ISP) and Butanone (BNA) were obtained from Sigma-Aldrich (St.Louis, CA, USA).

Buffer solutions used in this work were prepared as follows: 0.02 M potassium chloride (KCl pH 1.2) solution was made by dissolving 1.491 g of KCl in 1 L of Mili-Q water. 0.01 M phosphate-buffer saline (PBS pH 6.8) solution was prepared by dissolving 0.8 g of sodium chloride (NaCl), 0.02 g of KCl, 0.144 g of sodium phosphate dibasic (Na_2_HPO_4_) and 0.02 g of potassium phosphate monobasic (KH_2_PO_4_) in 1 L of Mili-Q water. The pH of the buffer solutions was adjusted using 37% hydrochloric acid (HCl) and diluted 1 M HCl, to reach pH values of 1.2 and 6.8, respectively.

### 2.2 Salt Synthesis

Before the mechanochemical synthesis, neutral **MTF** was obtained following the procedure previously described by our group.^12^

**PRX–MTF** and **TNX–MTF** were synthesized via Liquid-Assisted Grinding (LAG) using a Retsch MM2000 ball mill (Retsch, Haan, Germany) equipped with 10 mL stainless steel jars and two 5 mm stainless steel balls. Each ground mixture contained 0.2 mmol of the corresponding oxicam (66.27 mg of **PRX** or 67.47 mg of **TNX**), 0.2 mmol of **MTF** (25.83 mg), and 100 μL of solvent (DCM for **PRX–MTF**; ACE for **TNX–MTF**). The samples were milled at 25 Hz for 30 min.

**MLX–MTF** was obtained by slurry (SL) operations, in which 0.2 mmol of **MLX** (70.28 mg) and 0.2 mmol of **MTF** (25.83 mg) were suspended in 600 μL of DCM and stirred at 300 rpm at room temperature. After 24 h, the suspension was centrifuged at 10.000 rpm, the pellet was separated and dried at room temperature.

All bulk products were characterized by PXRD to confirm phase purity before any further solid-state characterization.

Single crystals of **PRX–MTF**, **MLX–MTF**, and **TNX–MTF** were obtained by slow evaporation of saturated solutions of the LAG or SL products in ACE, MET, and ETA, respectively. Crystals suitable for single-crystal X-ray diffraction (SCXRD) analysis were collected after 24–48 h.

### 2.3 Characterization Techniques

#### 2.3.1 X-ray Diffraction

PXRD patterns were recorded using a Bruker D8 Advance Series II Vario diffractometer (Bruker AXS, Karslruhe, Germany) equipped with a Ge(111) primary monochromator and a LynxEye fast silicon strip detector. The generator operated at 40 kV and 40 mA, using Cu Kα radiation. Measurements were performed in transmission geometry between Mylar foils with the beam focalized in the detector.

SCXRD data were collected at room temperature on a Bruker D8 Venture diffractometer (Bruker-AXS, Karlsruhe, Germany) using Cu Kα radiation (λ = 1.54178 Å). Crystals were handled under inert conditions using perfluoropolyether oil as protective oil. The data were processed with the software APEX4.^45^ The structure was solved with intrinsic phasing^46^ and refined with full-matrix least-squares on F^2^ ^47^ using Olex2 as a graphical interface.^48^ The non-hydrogen atoms were refined anisotropically. Hydrogen atoms were positioned in difference Fourier maps and included as fixed contributions riding on attached atoms with isotropic thermal displacement parameters 1.2 or 1.5 times those of the respective atom. Mercury,^49^ Platon^50^ and Olex2 ^48^ were used for visualization and graphical outputs. CIF files are deposited in the Cambridge Structural Database (CSD) under the CCDC numbers: 2500779–2500781. Copies of the data can be obtained free of charge at https://www.ccdc.cam.ac.uk/structures/ (accessed on 06 November 2025).

#### 2.3.2 Theoretical Methods

All quantum chemical calculations were performed using the Gaussian 16 program package.^51^ The PBE0 hybrid density functional,^52^ combined with Grimme’s D3 empirical dispersion correction,^53^ was employed for all electronic structure analyses. This level of theory, PBE0-D3/def2-TZVP, utilized the def2-TZVP basis set for all atoms.^54^ All calculations were based on the experimentally determined X-ray coordinates of the salts.

The Quantum Theory of Atoms in Molecules (QTAIM) analysis^55^ was conducted using the AIMAll program.^56^ This topological analysis of the electron density was used to characterize intermolecular interactions, including hydrogen bonds and C–H···π contacts. Noncovalent Interaction (NCI) plots^57^ were also generated using AIMAll at the same PBE0-D3/def2-TZVP level of theory, providing visual insights into the nature of these interactions.

The strength of the hydrogen bonds was estimated using the method proposed by Espinosa et al.,^58^ which correlates the interaction energy with the potential energy density (*V*(*r*)) at the bond critical point (BCP). Molecular Electrostatic Potential (MEP) surfaces were computed at the PBE0-D3/def2-TZVP level of theory, using an isovalue of 0.001 au, and subsequently visualized and plotted using GaussView.^59^

Molecular orbital calculations were performed using the Dmol3 program code (Accelrys, Inc.),^60^ employing the PBE functional^61,62^ with the double numerical with polarization (DNP) basis set^63^ in the all-electron scheme, Γ point set, and periodic boundary conditions on a single unit cell composed of four anions and four cations. This methodology was chosen to accurately reflect the solid-state electronic environment, as isolated formula units do not account for the structural constraints and intermolecular interactions that dictate fluorescence behavior in the crystal lattice.

#### 2.3.3 Fluorescence Studies

Solid-state fluorescence spectra were collected on a Cary Eclipse spectrophotometer (EL07023675 series) with scan software version 1.1 (132), in emission scan mode at a rate of 1200 nm/min. The data were collected from 400 to 700 nm. Excitation wavelengths and excitation/emission slit widths for each compound were measured as follows: **PRX** (365 nm, 2.5/2.5 nm), **PRX–MTF** (365, 2.5/2.5 nm), **MLX** (360, 2.5/2.5 nm), **MLX–MTF** (360, 2.5/2.5 nm and 5/5 nm), **TNX** (345, 2.5/2.5 nm) and **TNX–MTF** (345, 2.5/2.5 nm). Additionally, each compound was irradiated using a UCGL-25 UV lamp 4W (PL series) at two different wavelengths (254/365 nm).

#### 2.3.4 Thermal Analysis

Differential scanning calorimetry (DSC) and thermogravimetric analysis (TGA) studies were conducted using a Mettler-Toledo SC-822e calorimeter (Mettler Toledo, Columbus, OH, USA). The experimental conditions were: aluminum (Al_2_O_3_) crucibles of 40 μL volume, under a dry nitrogen atmosphere with 50 mL/min flow rate, heating rates of 1 °C/min and 10 °C/min. The calorimeter was calibrated with indium of 99.99% purity (m.p.: 156.4 °C; DH: 28.14 J/g).

#### 2.3.5 Thermodynamic Stability

The thermodynamic stability of the solid forms was assessed under accelerated aging conditions. Approximately 200 mg of each sample was placed in a watch glass and stored at 40 °C and 75% of relative humidity (RH) in a Memmert HPP110 climate chamber (Memmert, Schwabach, Germany). Samples were monitored by PXRD over a period of 2 months.

Stability in aqueous environments was assessed through SL experiments. An excess of solid was added to 1 mL of either (1) 0.02 M KCl buffer at pH 1.2 or (2) 0.01 M PBS buffer at pH 6.8. After stirring for 24 h at 25 °C in sealed vials, the solids were recovered, filtered, dried, and analyzed via PXRD.

#### 2.3.6 Solubility Studies

Powder dissolution profiles were determined for the pure oxicams and their corresponding PMMs using a Varian Cary 50 UV–Vis spectrophotometer (Agilent Technologies, Santa Clara, CA, USA). Standard calibration curves were established for **PRX**, **MLX**, and **TNX** at 358, 366, and 370 nm, respectively, to avoid interferences from **MTF** (maximum absorbance at 222 nm).

These experiments followed the shake-flask method,^64^ in which an excess of solid phase was added to 12 mL of either 0.01 M PBS buffer (pH 6.8) or 0.02 M KCl buffer (pH 1.2) and stirred at 25 °C for 24 h to reach thermodynamic equilibrium. At various time points (1, 2, 3, 4, 5, 6, 30 min and 1, 3, and 24 h), aliquots were withdrawn, filtered through 0.22 μm PES syringe filters, and analyzed by UV–Vis spectrophotometry. Dilutions were made as necessary to obtain absorbance within the calibration range.

## 3 Results and Discussion

### 3.1 Mechanochemical Synthesis

In this work, mechanochemical and semiliquid techniques were used to synthesize three novel oxicam**–**MTF materials. These methodologies have been selected due to their advantages in the screening of PMMs. LAG is a fast and solvent-efficient technique that enables rapid screening of solid-state forms by combining mechanical energy with small amounts of solvent, which act as additive promoting molecular diffusion and interaction. However, it is a kinetically controlled process and may fail to yield thermodynamically stable products, especially when energy barriers are high or molecular mobility is limited.^65,66^ In contrast, SL methods rely on the thermodynamic equilibrium between solid and solvent phases, making them more suitable for isolating the most stable solid forms. While SL reactions often require longer processing times and larger amounts of solvent, they can overcome kinetic limitations and are particularly useful when mechanochemical routes are unsuccessful.^67,68^ These complementary characteristics were leveraged in this study to explore a wider range of conditions for PMMs formation.

Initial grinding was conducted without the addition of any solvent, but these only resulted in the formation of physical mixtures of **MTF** with the respective oxicam. A wider screening of LAG conditions was carried out using a range of solvents, including mili-q H_2_O, MET, EOH, ETA, BNA, ACE, and DCM. Among the tested solvents, only DCM and ACE successfully promoted the formation of new crystalline phases: DCM enabled the synthesis of **PRX–MTF**, while ACE facilitated the formation of **TNX–MTF** (Figure 2).

**Figure 2:**
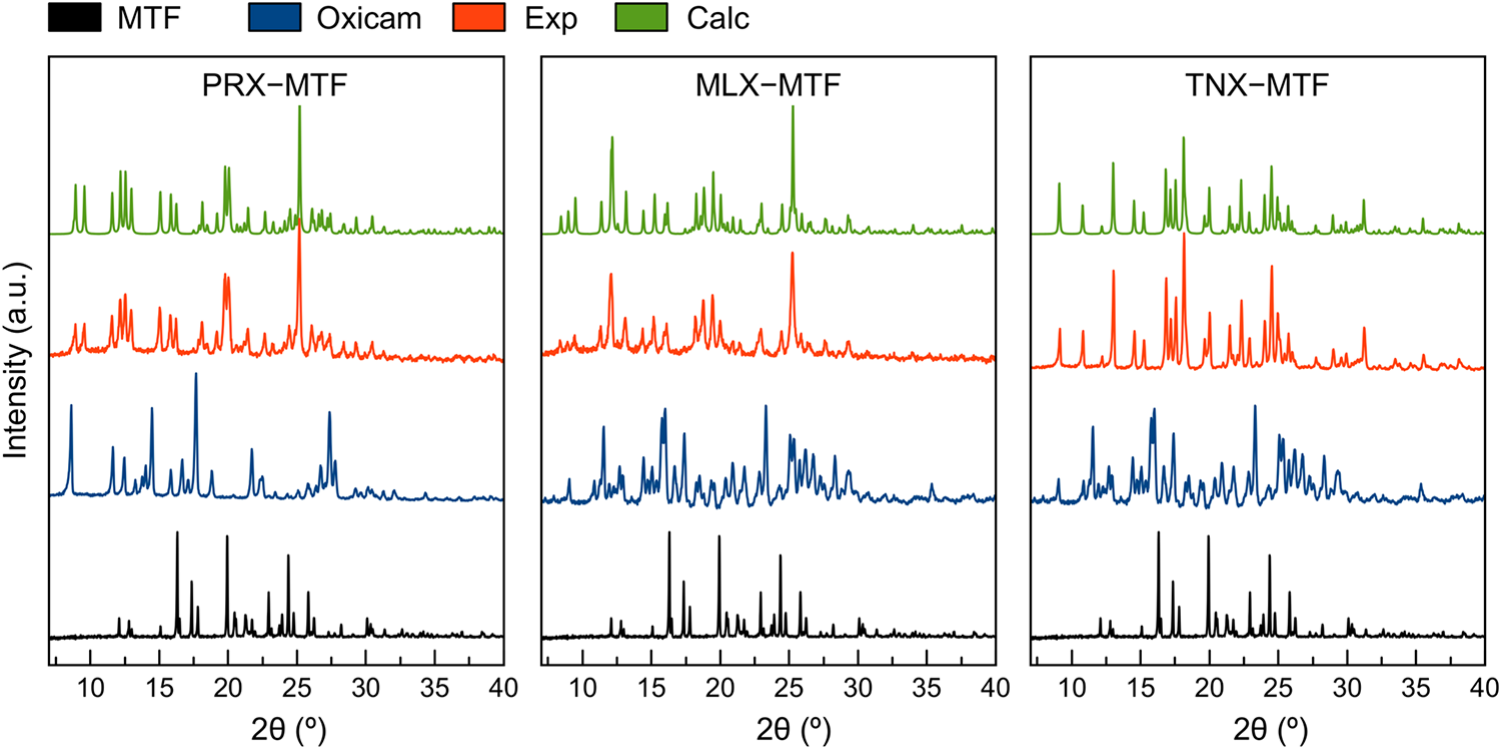
PXRD patterns of **MTF**, oxicams and the experimental and calculated products of the synthesis reactions.

Interestingly, no positive results were obtained for the **MLX–MTF** system via LAG. To avoid possible kinetic limitations, thermodynamically driven SL experiments were conducted, leading to the successful formation of a novel **MLX–MTF** phase when DCM was used. The PXRD pattern of **MLX** (with characteristic peaks at 12.8°, 13.3°, 14.7°, 18.5°, and 25.6°) and **MTF** were compared with the **MLX–MTF** product, confirming the formation of a third novel phase.

In addition to qualitative analysis, PXRD provides valuable information regarding the purity of the synthesized samples. The absence of diffraction peaks corresponding to the starting materials confirms the high purity of the bulk products.

After the mechanochemical synthesis, single-crystals suitable for structure determination were obtained via slow evaporation (2 days) of saturated solutions of the synthesis products, using ACE for **PRX–MTF**, MET for **MLX–MTF**, and ETA for **TNX–MTF**. In addition, the determination of the crystal structure allows the comparison of the experimental PXRD patterns with the calculated (Calc) PXRD patterns derived from the SXCRD analysis (Figure 2), providing a final confirmation of the phase purity and structural identity of the newly formed solid phases.

### 3.2 Crystal Structure Analysis

Salt or cocrystals formation is commonly rationalized using the “Δp*K*_a_ rule”, which relates the likelihood of proton transfer to the difference in p*K*_a_ values between the basic and acidic components.^69^ Extensive analyses have shown that Δp*K*_a_ values greater than 3–4 strongly favor salt formation, whereas Δp*K*_a_ < −1 strongly favors the formation of neutral cocrystals. For intermediate values (−1 < Δp*K*_a_ < 4), the degree of ionization must be carefully evaluated, as it depends on the overall crystal packing and the contribution of secondary intermolecular interactions.^70^

In this case, **PRX** (p*K*_a_ ≈ 5.1–5.3), **MLX** (p*K*_a_ ≈ 5.8–6.0), and **TNX** (p*K*_a_ ≈ 5.2–5.4) act as weak acids, whereas **MTF** is a strong base (p*K*_a_ > 12). Consequently, the Δp*K*_a_ values are greater than 6, pointing to the formation of Oxicams**–**MTF salts. SCXRD data allowed to confirm this hypothesis by confirming the proton transfer from the −OH group of oxicams to the primary amine group of **MTF**. A summary of the crystallographic data is provided in Table 1 at the end of this section, while the corresponding asymmetric units are illustrated in Figure 3.

**Figure 3:**
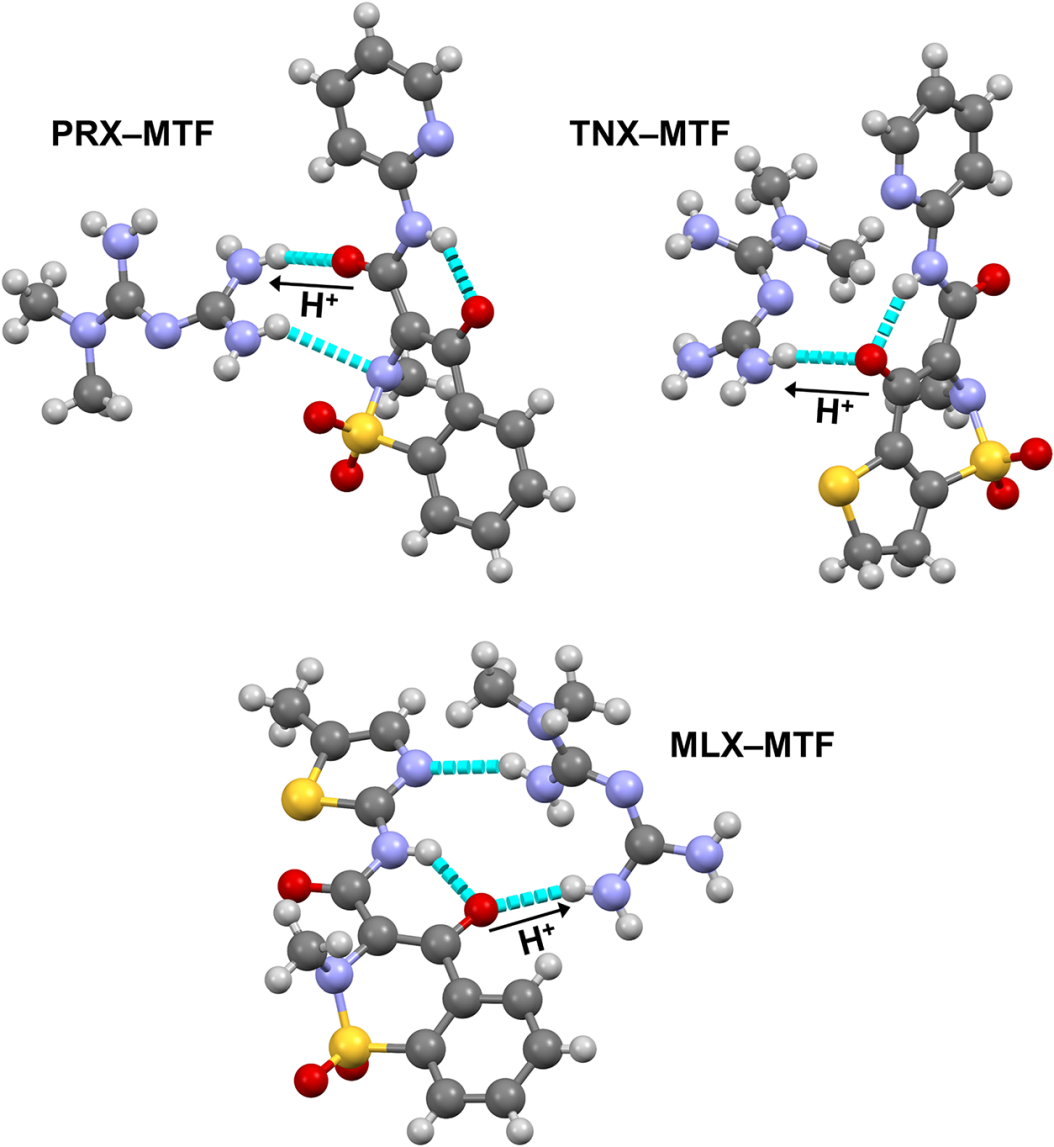
Asymmetric units of the oxicam**–**MTF salts presented in this work.

**Table 1:** Relevant Crystallographic Data and Structure Refinement Details of Oxicam–MTF Salts

compound name	**PRX–MTF**	**MLX–MTF**	**TNX–MTF**
formula	C_19_H_24_N_8_O_4_S	C_18_H_24_N_8_O_4_S_2_	C_17_H_24_N_8_O_4_S_2_
formula weight	460.52	480.57	468.56
crystal system	monoclinic	monoclinic	orthorhombic
space group	*P*2_1_/*c*	*P*2_1_/*c*	*Pna*2_1_
*a*/Å	10.0540(15)	10.4914(2)	12.1908(6)
*b*/Å	11.7334(17)	11.6033(2)	9.0492(5)
*c*/Å	18.486(3)	18.6772(4)	19.4314(8)
α/°	90	90	90
β/°	90.257(5)	94.222(10)	90
γ/°	90	90	90
*V*/Å^3^	2180.7(5)	2267.5(8)	2143.61(18)
*Z*	4	4	4
*D*_c_/g cm^–3^	1.403	1.408	1.452
*F*(000)	968	1008	984
reflections collected	17,487	27,205	47,972
unique reflections	3831	4015	3752
data/restraints/parameters	3831/0/293	4015/0/293	3752/1/284
goodness-of-fit on F2	1.053	1.033	1.041
*R*_1_ (*I* > 2σ(*I*))	0.0412	0.0338	0.0334
*w*R**_2_ (*I* > 2σ(*I*))	0.0973	0.0941	0.0883

**PRX–MTF** salt crystallizes in the monoclinic system, space group *P*2_1_/*c*, with one **PRX** ^**–**^ anion and one **MTF** ^**+**^ cation in the asymmetric unit, yielding a 1:1 stoichiometric salt. The ions are connected via N–H···O (alkoxy) hydrogen bonds between the hydroxyl group of **PRX** ^–^ and the terminal −NH_2_ group of **MTF** ^**+**^, and another hydrogen bond that involves one −NH_2_ group of **MTF** ^**+**^ and the C–NH–C pyridine group of **PRX** ^–^. These main interactions are described by the *R*_2_^2^(14) graph set, generating dimers (Figure 4a).

**Figure 4:**
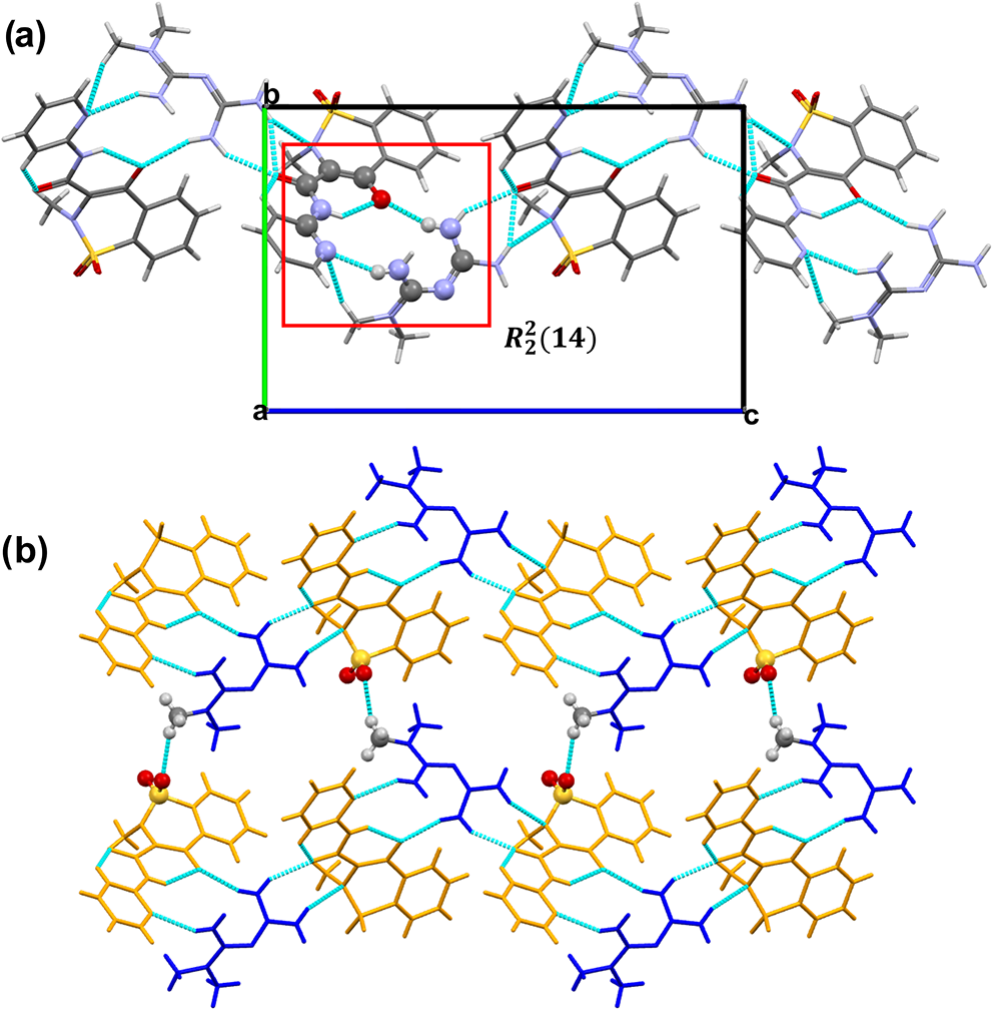
(a) View along the *c*-axis highlighting the one-dimensional zigzag chains observed in **PRX**–**MTF**. Red box indicates the graph set of **PRX**–**MTF**, whose atoms are represented as balls, while the rest of the atoms are represented as sticks. (b) Detailed view of the bidimensional layered structure of **PRX**–**MTF**. **MTF** ^+^ cations are represented in blue while **PRX** ^–^ anions are represented in yellow. Atoms involved in the interactions between chains are represented as standard balls, while the rest of the atoms are represented as sticks.

Similar hydrogen-bonding patterns have been reported for other oxicam salts formed with amine-containing molecules.^43^ Notably, analogous heterosynthons are also observed in oxicam cocrystals with acidic coformers; however, in those cases the proton donor is the acidic coformer (−OH) and the proton acceptor is the oxicam molecule.^71–73^ This highlights the strong and thermodynamically favorable nature of the N–H···O interaction across the oxicam multicomponent materials family.

In **PRX–MTF** these hydrogen bond interactions are supported by additional intramolecular H-bonds between the N–H···H–O of **PRX** ^–^ and another N···H–C interaction between the Pyridine group of **PRX** ^–^ and the terminal −CH group of **MTF** ^**+**^. This set of interactions allows to stabilize the salt. The dimeric units are further connected by C=O···H–N hydrogen bonds between the ketone group of **PRX** ^**–**^ and **MTF** ^**+**^, using the free H atom of the −NH_2_ group that is also involved in the already mentioned *R*_2_^2^(14) graph set. Moreover, this C=O group is engaged in an intramolecular interaction with a – CH group of the aromatic ring of **PRX** ^**–**^ providing stability. An additional set of H-bonds between the H–N group of **MTF** ^**+**^ with both the ketone and −NH group close to the sulfone (SO_2_) group of **PRX** ^**–**^ results in a shared hydrogen bond (Figure 4a), allowing the union of the dimeric pairs, and resulting in the formation of zigzag molecular chains along the *c*-axis (Figure 4a). The one-dimensional zigzag chains are further linked through S=O···CH interactions, giving rise to two-dimensional sheet-like assemblies as shown in Figure 4b. The final three-dimensional supramolecular framework is constructed via π–π stacking between the aromatic regions of adjacent **PRX** ^–^ molecules.

**MLX**–**MTF** salt shares similar crystallographic features with the **PRX**–**MTF** system. It crystallizes in the monoclinic system with *P*2_1_/*c* space group, and its asymmetric unit also comprises one **MLX** ^–^ anion and one **MTF** ^**+**^ cation in a 1:1 ratio. Additionally, the main interactions that connect the ionic pairs are also described by the by the *R*_2_^2^(14) graph set: (1) N–H···H–O hydrogen bonds between the hydroxyl group of **MLX** ^**–**^ and the terminal – NH_2_ group of **MTF** ^+^; (2) N–H···H–N, involving the −NH_2_ group of **MTF** ^**+**^ and the SO_2_–C–NH motif of **MLX** ^**–**^. These ionic dimers are stabilized by an additional intramolecular H-bond between the N–H···H–O of **MLX** ^–^ and another N···H–C interaction between the SO_2_–C–NH motif of **MLX** ^–^ and the terminal – CH group of **MTF** ^**+**^. This set of interactions stabilizes the ionic dimers, as shown in Figure 5a. These dimers are further connected to form one-dimensional chains along the *b*-axis via C–H···O–S interactions. However, a key difference lies in the spatial disposition of the **MTF** ^**+**^ molecule relative to the oxicam. While in **PRX**–**MTF**, the **MTF** ^**+**^ is in the same plane of the oxicam, in **MLX–MTF**, the **MTF** ^**+**^ cation adopts a nearly perpendicular orientation (82.11°) which leads to the folding of the 1D chains (Figure 5b).

**Figure 5:**
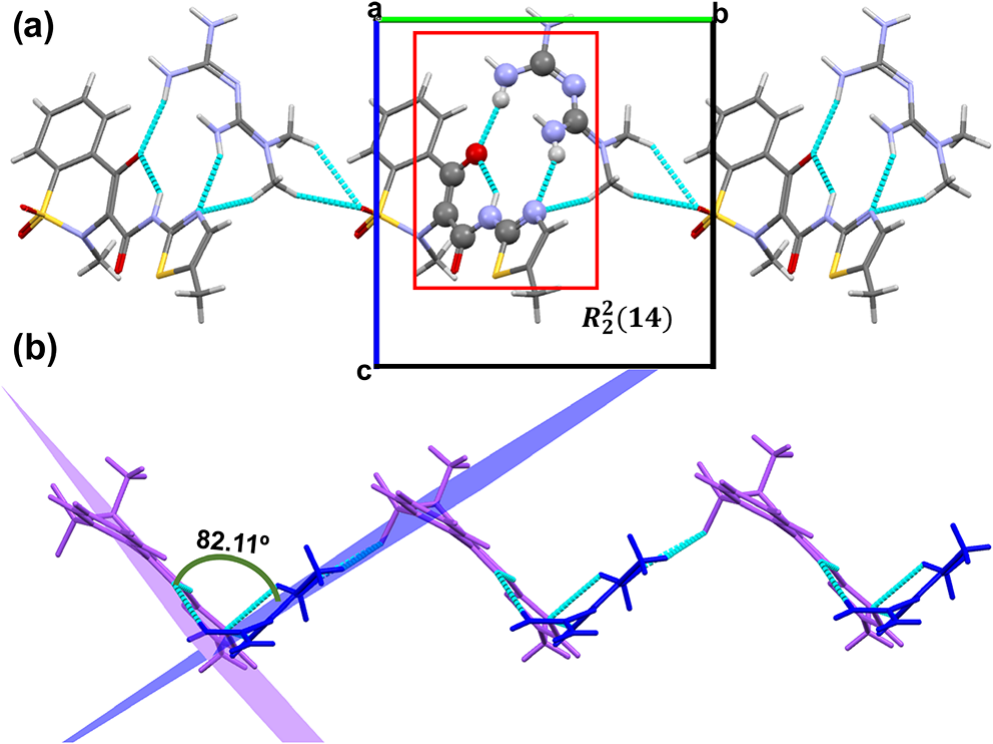
(a) View along the *b*-axis highlighting the one-dimensional chain observed in **MLX**–**MTF**. Red box indicates the graph set of **MLX**–**MTF**, whose atoms are represented as balls, while the rest of the atoms are represented as sticks. (b) Detailed view of the folded chain structure of **MLX**–**MTF**. **MTF** ^+^ cations are represented in blue, while **MLX** ^–^ anions are represented in purple.

These one-dimensional chains associate laterally to form bidimensional folded-sheets through a network of intermolecular hydrogen bonds. Specifically, N–H···O–H interactions occur between the terminal −NH_2_ groups of **MTF** ^+^ and the hydroxyl groups of **MLX** ^–^. Additionally, further stabilization arises from N···H–N hydrogen bonds involving the nitrogen atom adjacent to the SO_2_ group of **MLX** ^–^ and another −NH_2_ group of **MTF** ^+^ (Figure 6a). Finally, these 2D sheets stack into a three-dimensional supramolecular architecture through C–H···π interactions, where alkyl hydrogen atoms interact with the aromatic rings of adjacent molecules. This π-stacking pattern contributes to the overall cohesion of the crystal structure, as illustrated in Figure 6b.

**Figure 6:**
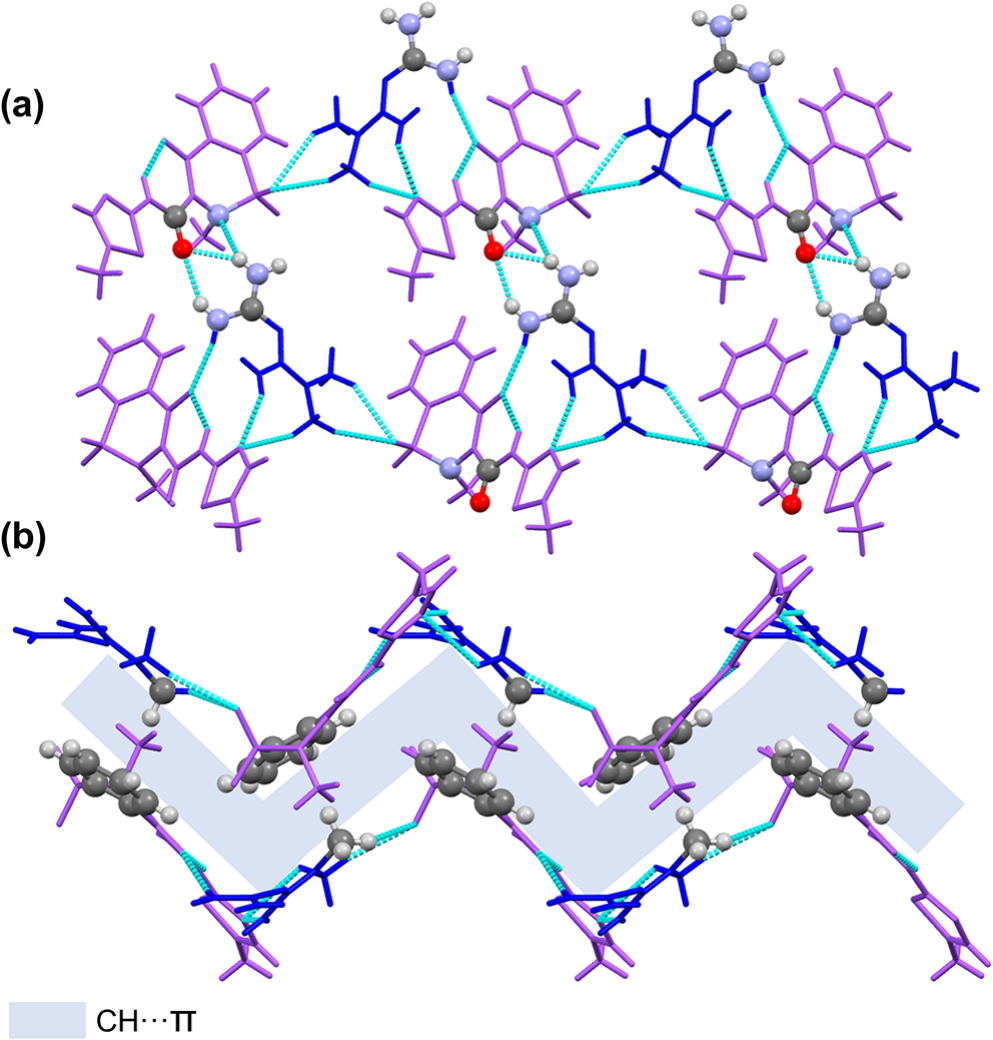
(a) Detailed view of the bidimensional layered structure of **MLX**–**MTF**. (b) Detailed view of the 3D stacking observed in **MLX**–**MTF**. **MTF** ^+^ cations are represented in blue, while **MLX** ^–^ anions are represented in purple. Atoms involved in the interactions between chains are represented as standard.

Among the studied salts, the **TNX**–**MTF** structure exhibits the most complex supramolecular organization. **TNX**–**MTF** salt crystallizes in the orthorhombic system with space group *Pna*2_1_. The asymmetric unit comprises one **TNX** ^–^ anion and one **MTF** ^+^ cation in a 1:1 ratio. In this system, a single **MTF** ^+^ cation bridges two **TNX** ^–^ anions, forming a supramolecular trimer. Unlike the previously described salts where **MTF** ^+^ is symmetrically positioned between two oxicam units, in this case both **TNX** ^–^ anions are located on the same side of the **MTF** ^+^ cation. This configuration results in the formation of two distinct hydrogen-bonding motifs: *R*_2_^2^(14) and *R*_2_^2^(11) graph sets (Figure 7a). The *R*_2_^2^(14) graph set is formed through two hydrogen bonds: one between the −NH_2_ group of **MTF** ^+^ and the hydroxyl group of **TNX** ^–^, and another involving one of the −NH_2_ of **MTF** ^+^ and the C–N–C moiety of the **TNX** pyridine ring. In the *R*_2_^2^(11) graph set, the hydrogen bonds involve the C=O of **TNX** ^–^ and an −NH_2_ group of **MTF** ^+^, along with an additional interaction between the nitrogen atom adjacent to the SO_2_ group of **TNX** ^–^ and another −NH_2_ group of **MTF** ^+^. The coexistence of these motifs leads to a more compact hydrogen-bond network, which correlates with the higher packing density observed in this structure (Table 1). Additional secondary interactions reinforce each graph set. In the *R*_2_^2^(14) motif, stabilization is enhanced by an intramolecular O–H···N hydrogen bond within the **TNX** ^–^ anion, as well as a C–H···N interaction involving the pyridine ring. In the *R*_2_^2^(11) motif, further stabilization arises from an intramolecular O–H···N contact within **TNX** ^–^ and an N–H···O=S hydrogen bond between the **MTF** ^+^ amino group and the sulfone group of **TNX** ^–^.

**Figure 7:**
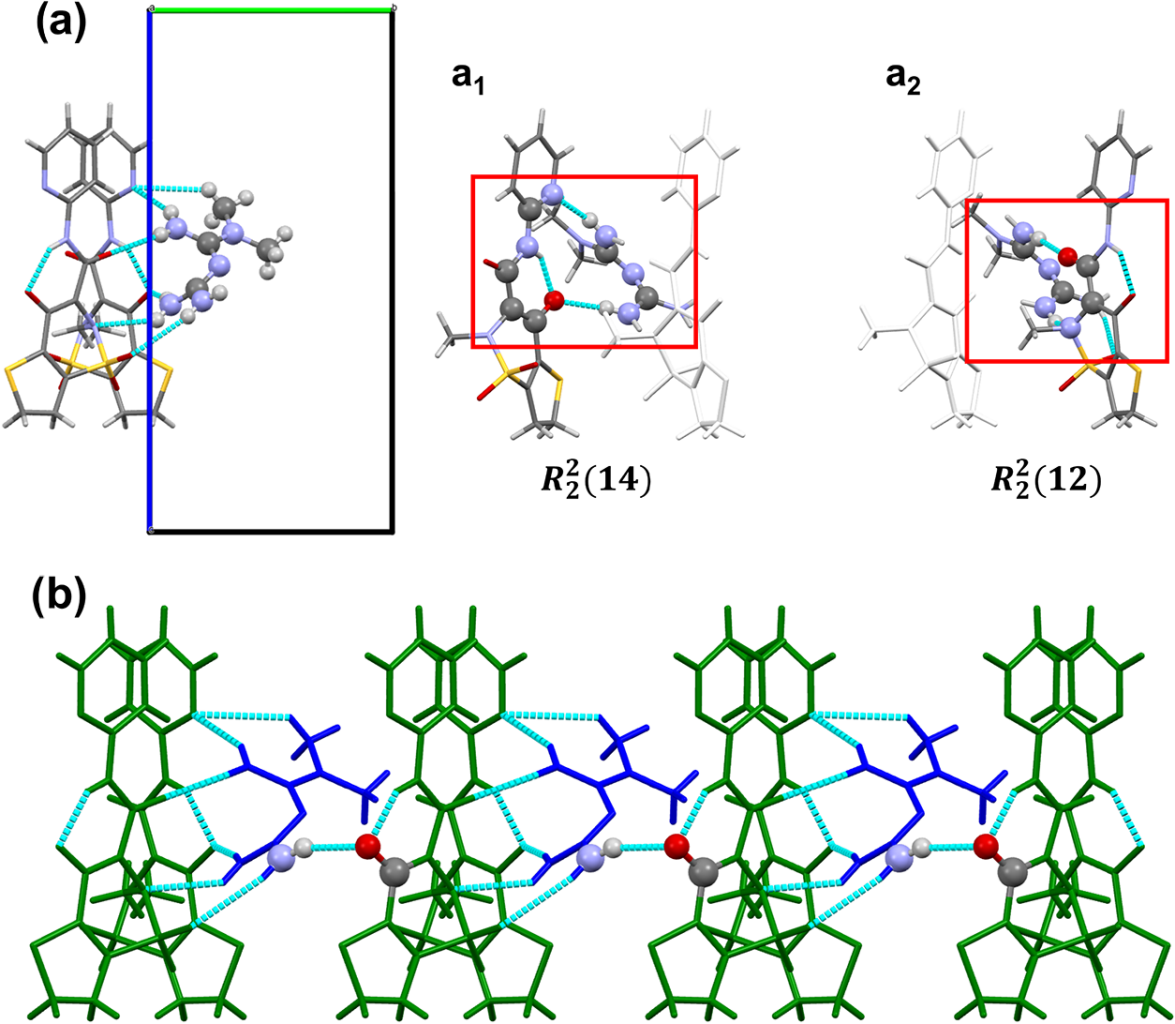
(a) Molecular trimer observed in **TNX–MTF**. For clarity, this view is expanded into (a_1_) and (a_2_), which represent the two observed graph-set motifs, *R*_2_^2^(14) and *R*_2_^2^(12), respectively. In each case, the molecules involved in the interaction are shown in color, while those not involved displayed in white. Atoms involved in the graphs set are represented as balls, while the rest are represented as sticks. (b) View along the *a*-axis, highlighting the one-dimensional chain observed in **TNX**–**MTF**. **MTF** ^+^ cations are represented in blue, while **TNX** ^–^ anions are represented in green. Atoms involved in the interactions between trimers are represented as standard balls, while the rest of the atoms are represented as sticks.

These supramolecular trimers further associate into one-dimensional chains along the *b*-axis, primarily through N–H···O hydrogen bonds between an −NH_2_ group of **MTF** ^+^ and the carbonyl oxygen involved in the intramolecular interaction of **TNX** ^–^. The 1D chains laterally associate to form 2D sheets, which in turn stack into a three-dimensional crystal structure. This hierarchical organization is stabilized by C–H···O=S contacts involving the sulfone group of **TNX** ^–^, as depicted in Figure 8, rather than the already described C–H···π and π···π interactions of **PRX**–**MTF** and **MLX**–**MTF**.

**Figure 8:**
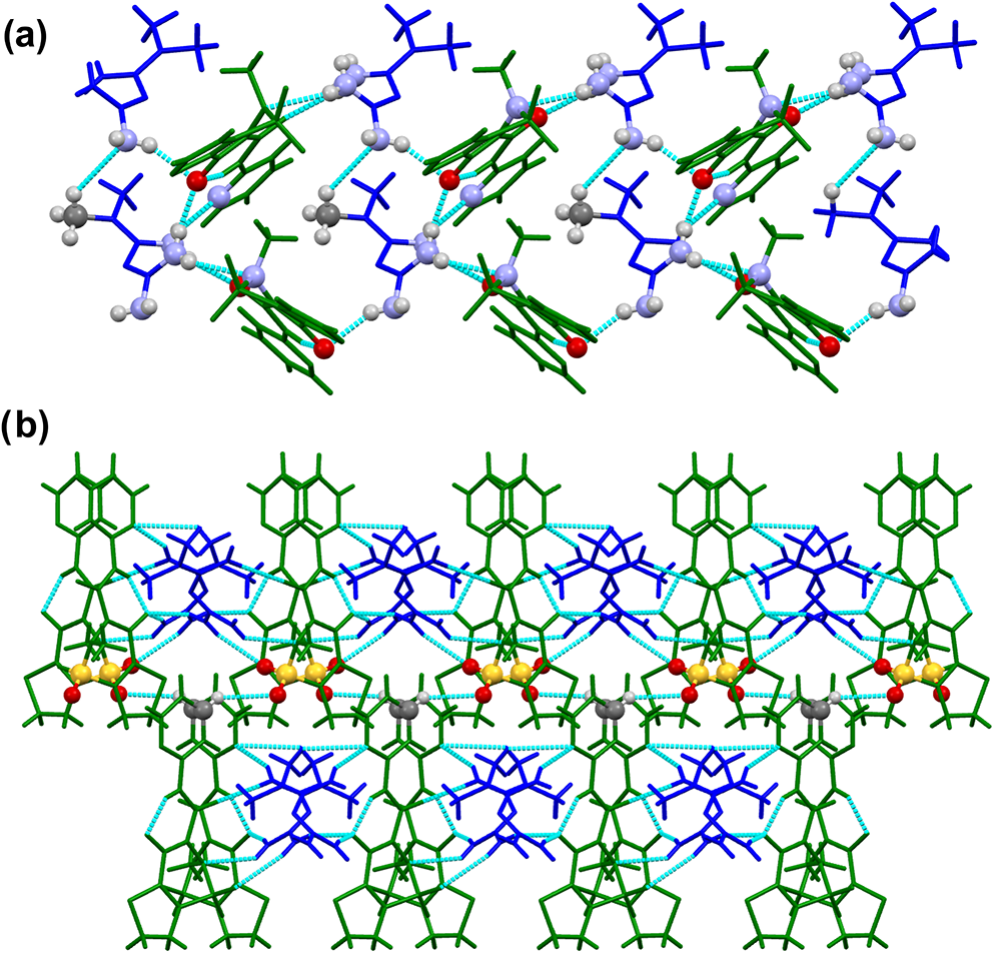
(a) Detailed view of the bidimensional layered structure of **TNX**–**MTF**. (b) 3D structure of **TNX**–**MTF**. **MTF** ^+^ cations are represented in blue, while **TNX** ^–^ anions are represented in green. Atoms involved in the interactions between chains are represented as standard balls, while the rest of the atoms are represented as sticks.

### 3.3 Density Functional Theory Analysis

DFT was performed as a complementary study to enhance the understanding of the intricate hydrogen–bonding networks and diverse supramolecular synthons observed in the crystal structures of the novel oxicam–MTF salts. A key aspect of our theoretical investigation involves the precise estimation of hydrogen bond strengths. Rather than relying solely on the traditional supramolecular approach, we have specifically utilized QTAIM analysis to quantify the energies of these hydrogen bonds. This methodology allows us to effectively isolate the hydrogen–bonding component by avoiding the confounding contributions of pure electrostatic (Coulombic) forces, thereby providing a more accurate assessment of their strengths within the crystal lattice. Furthermore, our study has also explored intriguing C–H···π interactions observed in **PRX–MTF** and **MLX–MTF**, adding another layer to the understanding of their supramolecular assembly.

Figure S1 displays the calculated MEP surfaces for (a) the cationic **MTF** ^+^, and the anionic forms (b) **PRX** ^–^, (c) **MLX** ^–^, and (d) **TNX** ^–^. These surfaces provide insights into the charge distribution and potential sites for intermolecular interactions. In the case of the **MTF** ^+^, the entire MEP surface is positive, consistent with its protonated and high reactivity nature. The minimum positive potential is located at the central nitrogen atom (58.9 kcal/mol), while the maximum positive potential (138.0 kcal/mol) is observed between the two terminal NH_2_ groups. Other significant positive MEP values are also found at H–atoms of the remaining NH_2_ group (127.4 and 125.5 kcal/mol). These highly positive regions clearly indicate the excellent hydrogen bond donor ability of **MTF** ^+^ and supports the proton transfer and strong N–H···O interactions described in the crystal structure analysis.

The three anionic compounds (**PRX** ^–^, **MLX** ^–^, and **TNX** ^–^) exhibit similar MEP characteristics, highlighting their consistent hydrogen bond acceptor capabilities. The most negative MEP regions (minima) are consistently located at the amidic oxygen atom, with values ranging from −117.3 kcal/mol (**MLX** ^–^) to −118.6 kcal/mol (**TNX** ^–^). The hydroxide oxygen atom also presents significant negative potentials, ranging from −108.6 kcal/mol (**MLX** ^–^) to −114.2 kcal/mol (**TNX** ^–^). Additionally, the oxygen atoms of the sulfone group show large negative MEPs, ranging from −87.8 kcal/mol (**PRX** ^–^) to −107.9 kcal/mol (**MLX** ^–^). The nitrogen atoms of the pyridine or thiazole rings also exhibit considerable negative MEP values, ranging from −100.4 kcal/mol (**MLX** ^–^) to −104.8 kcal/mol (**TNX** ^–^). These pronounced negative regions across multiple sites within the anions confirm their strong hydrogen bond acceptor ability, which is crucial for explaining the extensive hydrogen–bonding networks observed in the solid–state structures of the three salts.

Figure S2 displays the QTAIM analysis of three distinct dimeric assemblies extracted from the X–ray structures of **PRX–MTF** (top row: a, b, c) and **MLX–MTF** (bottom row: d, e, f). Only intermolecular BCPs and bond paths are represented for clarity. BCPs for hydrogen–bonding interactions are shown as green spheres, while those for C–H···π contacts are red, differentiating them from the green RDG (Reduced Density Gradient) isosurfaces used to visualize π–interactions. As described in the crystal structure analysis, both salts exhibit hydrogen–bonded dimers and C–H···π contacts, consistent with their similar crystal packing architecture.

The first type of dimer, depicted in panels (a) and (d), features a combination of an N–H···O­(alkoxy) hydrogen bond and an *R*_2_^2^(7) supramolecular ring involving one N–H and one C–H bond as donors. The total hydrogen–bonding energies for these dimers are −7.1 kcal/mol for **PRX**–**MTF** and −7.5 kcal/mol for **MLX**–**MTF**. The *R*_2_^2^(7) supramolecular ring, which includes a bifurcated hydrogen bond, is observed to be stronger than the N–H···O­(alkoxy) hydrogen bond.

The second type of dimer, shown in panels (b) and (e), is formed by two fused supramolecular rings: *R*_2_^1^(6) and *R*_2_^1^(5). Notably, the *R*_2_^1^ (6) ring is formed between what are identified by MEP surface analysis as the best hydrogen–bond donor and hydrogen–bond acceptor groups. Agreeably, this corresponds to the stronger synthon within this dimeric motif, with energies of −6.1 kcal/mol for **PRX**–**MTF** and −5.9 kcal/mol for **MLX**–**MTF**. The energies of the *R*_2_^1^(5) rings are −4.1 kcal/mol for **PRX**–**MTF** and −4.3 kcal/mol for **MLX**–**MTF**, similar to those of the *R*_2_^1^(7) supramolecular rings. The total energies of these second dimers are −7.3 kcal/mol for **PRX**–**MTF** and −6.8 kcal/mol for **MLX**–**MTF**. When considered in conjunction with other dimeric interactions, these represent a total interaction energy from the hydrogen–bonding network of −14.4 kcal/mol for **PRX**–**MTF** and −14.3 kcal/mol for **MLX**–**MTF**, respectively, indicating almost equivalent stabilization in both salts due to hydrogen bonding.

Finally, panels (c) and (f) illustrate the C–H···π interaction. This interaction is characterized by a BCP and bond path connecting the hydrogen atom of the methyl group of metformin to the phenyl ring of the anion. The π–nature of the interaction is identified by the NCIplot analysis, which displays an extended green RDG isosurface embracing the entire π–system. The interaction energies for these C–H···π contacts are also very similar for both salts, at −2.0 kcal/mol for **PRX–MTF** and −2.1 kcal/mol for **MLX–MTF**. The near–identical hydrogen–bond and C–H···π energies observed are consistent with the similar overall crystal packing architectures of both salts.

Figure S3 displays the QTAIM analysis of three distinct dimeric assemblies extracted from the **TNX–MTF** salt, which exhibits a different crystal packing compared to the other two salts previously discussed. In this case, all three dimers feature hydrogen bonds, with the third dimer also showing an ancillary C–H···π­(C=C) interaction involving a double bond. For this third dimer, NCIplot analysis has also been included, and hydrogen bond BCPs are shown in red for clarity when the NCIplot is present.

The first dimer, shown in panel (a), is characterized by two moderately strong hydrogen bonds: an N–H···O­(alkoxy) and an N–H···N­(pyridine) interaction. These contribute to a total dimerization energy of −8.5 kcal/mol.

In the second dimer, panel (b), three weaker hydrogen bonds are formed, with individual strengths ranging from −0.8 to −2.2 kcal/mol, resulting in a dimerization energy of −4.7 kcal/mol.

Finally, the third dimer, panel (c), presents a moderately strong hydrogen bond (−3.6 kcal/mol), characterized by a blue RDG isosurface from the NCIplot analysis. Additionally, a weak C–H···π interaction is present, identified by a BCP and bond path connecting a hydrogen atom of an NH2 group to a carbon atom of the C=C double bond in the anion. The π-nature of this interaction is visualized by a green RDG isosurface that occupies the entire region between the double bond π-system and the hydrogen atom. This C–H···π interaction is very weak (−0.6 kcal/mol), indicating that the overall dimerization in this motif is dominated by the stronger hydrogen bond.

Considering all three dimers, the total hydrogen bond energy in the **TNX–MTF** is −16.8 kcal/mol, which is approximately 2 kcal/mol more favored than in the **PRX–MTF** and **MLX–MTF** salts. This energetic difference in hydrogen bonding is, however, compensated by the C–H···π interactions, which are observed to be stronger in the **PRX**–**MTF** and **MLX**–**MTF** salts. Interestingly, the relative hydrogen-bond strengths derived from QTAIM follow the same trend indicated by the SCXRD packing densities. Taken together, the DFT and QTAIM results closely align with the crystallographic data, providing a quantitative foundation for the crystal structure description.

### 3.4 Fluorescence

Although oxicam molecules are known for their fluorescence properties,^74–76^ salt formation is known for completely disturbing the intermolecular interaction networks of the parent APIs, which influences the local environment of the emissive molecule.^77–79^ For this reason, the solid-state fluorescence emission spectra of the three salts have been analyzed, with the ultimate goal of demonstrating the influence of crystal packing, π–π interactions, and hydrogen-bond networks on their fluorescence behavior.

The first step in the fluorescence study involves identifying the regions of highest luminescence activity within the molecules, typically associated with the HOMO and LUMO orbitals. These orbitals provide insight into the electronic transitions responsible for light emission. Figure 9 shows the HOMO and LUMO orbitals observed for the three salts, that were computed using an unit cell composed by four anions and four cations and PBC conditions. In the case of **PRX–MTF** and **MLX–MTF**, the HOMO is predominantly localized in the enolic group of the oxicam moiety, which is expected given the similarity in their crystal structures. In contrast, for **TNX–MTF**, the HOMO is located in the five-membered ring, suggesting a different electronic environment. The HOMO–LUMO gap is also a key factor in understanding the emission energy, as it determines the energy (and thus the wavelength) of the emitted light. While the gaps across the three systems are relatively similar, **TNX–MTF** presents the smallest energy gap, mainly due to the implication of the five-membered ring in the fluorescence. This lower energy gap can also be associated with a higher wavelength of the emission fluorescence or broadened emission relative to the others.

**Figure 9:**
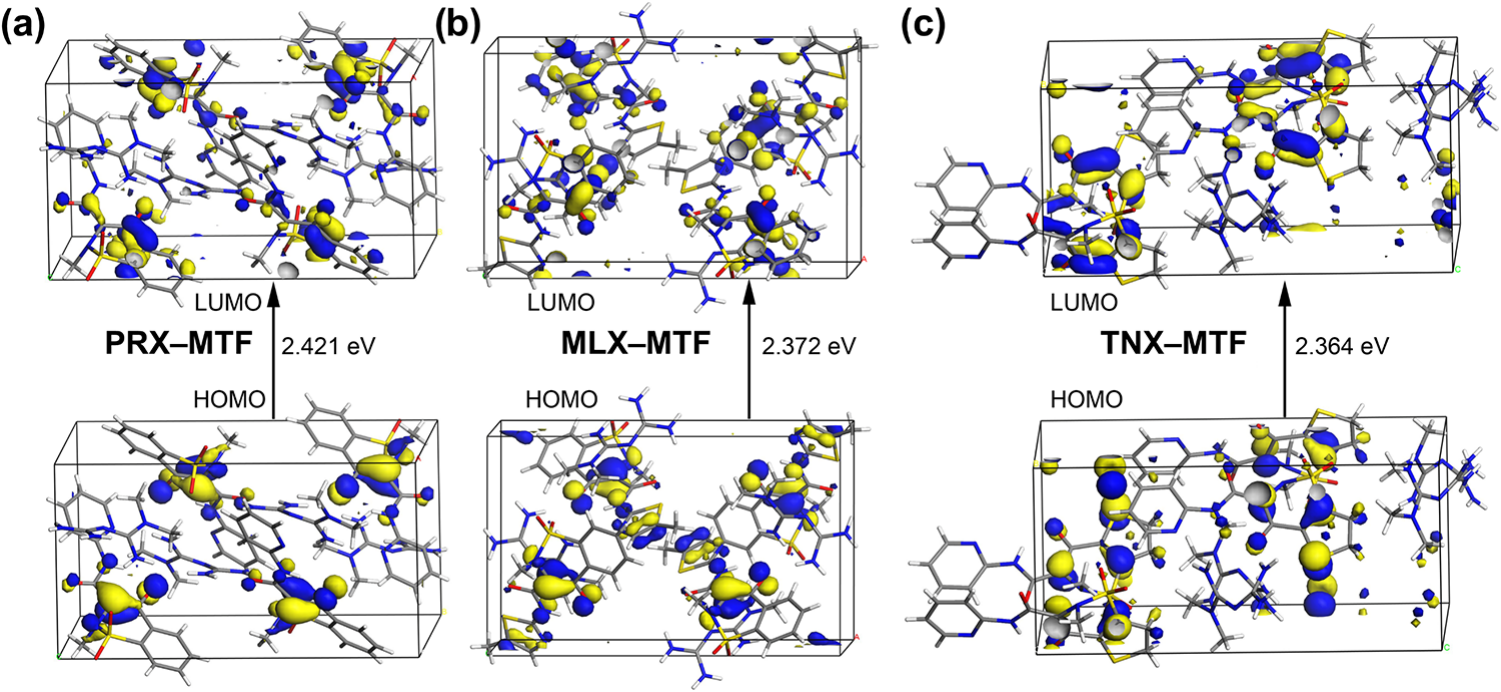
HOMO and LUMO orbitals of (a) **PRX–MTF** (b) **MLX–MTF** and (c) **TNX–MTF**, at the PBE/DNP level of theory.

Emission spectra (Figure 10a) confirm these trends. For **PRX–MTF**, the emission maximum shifts from 450 nm in **PRX** to 483 nm in the salt, accompanied by peak broadening. The calculated HOMO is predominantly localized on the enolic group of **PRX**, which is directly involved in the *R*_2_^2^(14) synthon and participates in π–π interactions within the crystal lattice. The slipped stacking geometry and relatively weaker hydrogen-bond network allow greater excited-state structural relaxation, facilitating lower-energy electronic transitions. This behavior is consistent with a J-type aggregate–like arrangement, where reduced electronic coupling promotes red-shifted emission.^80,81^

**Figure 10:**
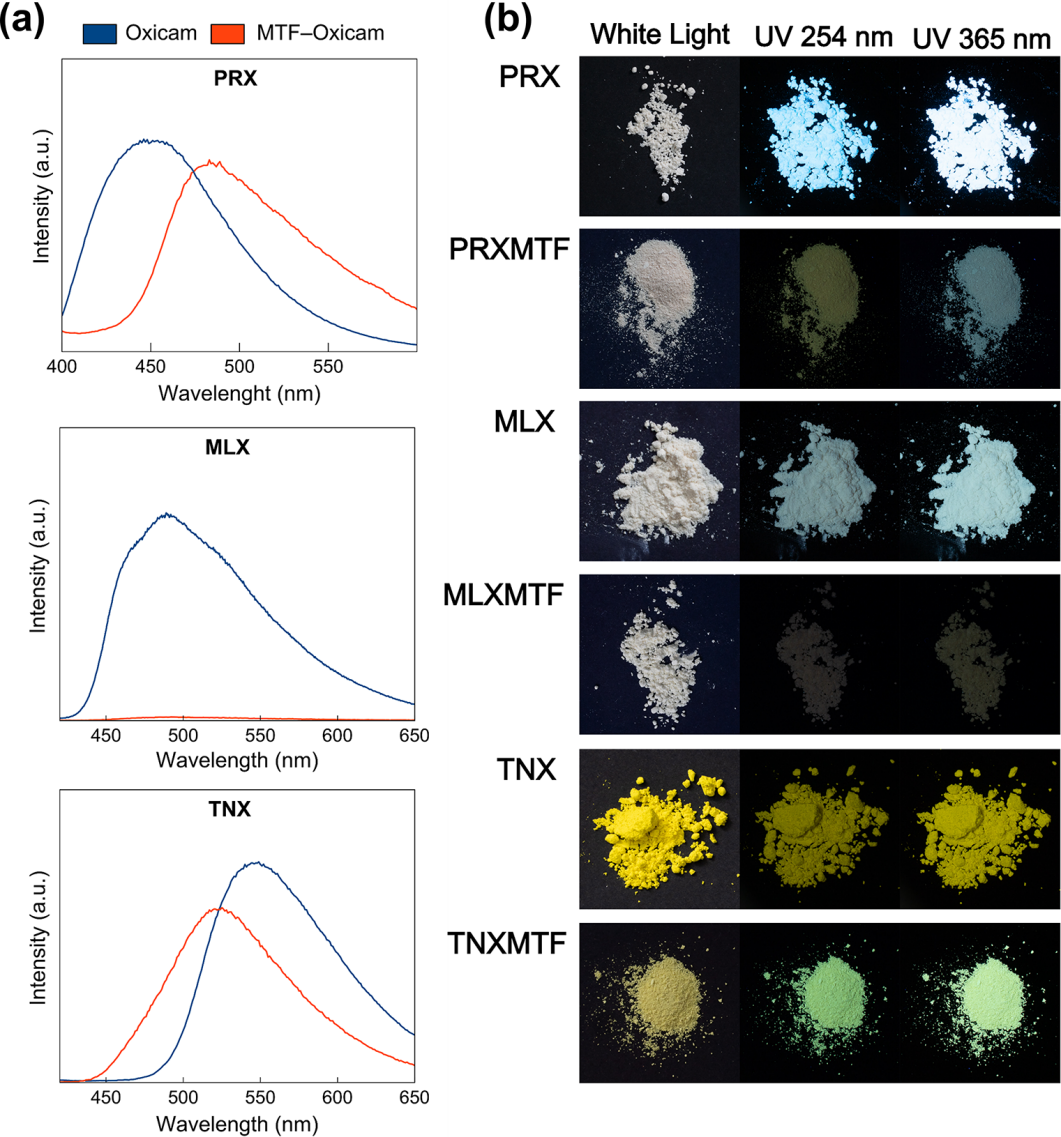
(a) Solid-state fluorescence emission spectra of **PRX–MTF**, **MLX–MTF**, **TNX–MTF** and the corresponding oxicam. (b) Photographs of the solid samples under UV irradiation (254 and 365 nm) using dark background.

Additionally, Figure 10b shows the visible emission of the solid samples when exposed to UV-light (254 and 365 nm) under dark background. The green emission observed under UV irradiation for **PRX–MTF** correlates well with the calculated HOMO–LUMO distribution and the maximum fluorescence peak observed for the salt.

Despite its structural similarities to **PRX–MTF**, **MLX–MTF** displays a markedly different fluorescence behavior. As shown in Figure 10b, the powder form of **MLX** emits green light under UV irradiation, consistent with its spectral maximum, yet **MLX–MTF** shows complete fluorescence quenching under the same conditions. In many conventional systems, highly emissive fluorophores often undergo partial or complete quenching upon aggregation, a phenomenon known as aggregation-caused quenching (ACQ), which arises from excited-state energy transfer in the solid state.^82–84^ Although **PRX–MTF** and **MLX–MTF** share similar hydrogen-bond motifs in their crystal frameworks, a key distinction lies in the spatial arrangement of the **MTF** ^+^ cation. In **MLX–MTF**, the cation is oriented nearly perpendicular (∼82°) to the enolic plane of **MLX** ^–^, a geometry that appears to facilitate nonradiative deactivation processes. The theoretical model of the unit cell was crucial in identifying this 82.11° orientation of the **MTF** ^+^ cation. While the orbital localization is similar to **PRX–MTF**, this specific geometric arrangement promotes the nonradiative deactivation pathways responsible for the observed complete fluorescence quenching This geometry does not correspond to a well-defined H- or J-type aggregate but rather to an intermediate aggregation mode commonly observed in multicomponent materials. This structure–property relationship is consistent with previous observations in analogous molecular salt systems.^43,85,86^

In addition, to assess whether the quenching phenomenon in **MLX–MTF** is complete, the fluorescence spectrum was recorded using wider slit widths (5/5 nm) (Figure S4). Under these conditions, an emission profile could be detected; however, it was accompanied by a high level of instrumental noise, indicating a very weak emission intensity and a pronounced effect of aggregation-caused quenching. Notably, despite the low emission intensity, no spectral shift was observed when compared with **MLX**.

Finally, the **TNX–MTF** salt presents a downshift in the maximum emission wavelength (524 nm) compared to **TNX** (545 nm). However, among the three salts, it displays the highest emission maximum. Additionally, the emission profile of the salt is broader, showing emission over a wider wavelength range than **TNX**. The spectral downshift is reflected in the visible emission shown in Figure 10b, where **TNX** emits light close to yellow, while the salt emits light closer to bright green. **TNX–MTF** possesses the smallest HOMO–LUMO energy gap (2.364 eV) among the studied salts. In this case, the HOMO is mainly localized on the five-membered ring rather than on the enolic group, which modifies the nature of the electronic transition and accounts for the intense green emission observed experimentally. Moreover, the presence of strong and directional hydrogen-bond interactions leads to a highly rigid crystal structure that restricts excited-state structural relaxation. This reduced relaxation limits energy dissipation through nonradiative pathways and results in higher-energy (blue-shifted) emission. Overall, this behavior is consistent with an H-type aggregate–like arrangement in the solid state.^80,81^

### 3.5 Thermal Stability

DSC-TGA investigated the thermal behavior of the salts, and compared with their corresponding APIs, including **MTF·HCl**. The DSC traces obtained in these studies are shown in Figure 11 along with the melting point of the APIs.

**Figure 11:**
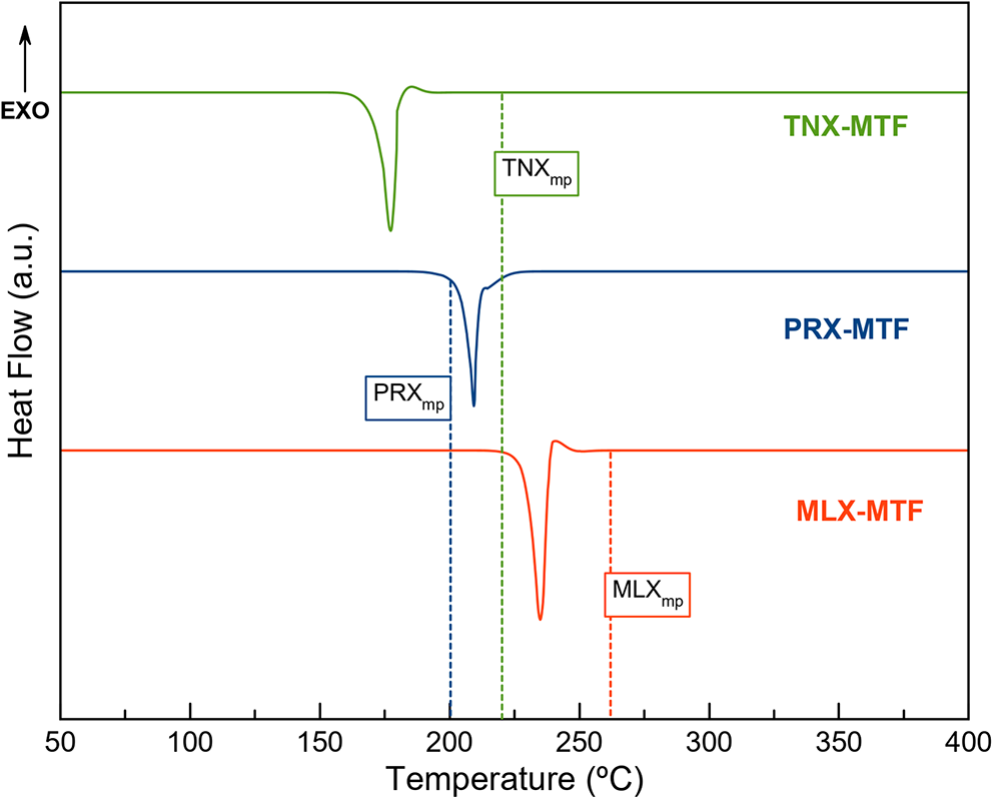
DSC traces of **PRX–MTF**, **MLX–MTF**, **TNX–MTF**. Dotted lines correspond to the reported melting point of the isolated APIs used in this work.

In all cases, only a major endothermic peak was observed, ascribed to the melting point of the materials. The absence of weight loss before the melting point in the corresponding TGA profiles (Figure S5) confirms that these events are not related to hydration or solvent loss and further supports the purity and anhydrous nature of the samples. For a better comparison, the melting points of all studied materials are summarized in Table 2.

**Table 2:** Melting Points of the APIs and Their Respective PMMs, Arranged by the Oxicam Melting Point

oxicam	melting point (°C)	salt	melting point (°C)
**PRX**	198–200	**PRX–MTF**	209.5
**TNX**	216–218	**TNX–MTF**	177.6
**MLX**	254–256	**MLX–MTF**	235.1
**MTF**	117	**MTF·HCl**	231.5

It is well established that the melting point of a salt or cocrystal often lies between those of its individual components.^87^ In this case, neutral **MTF** exhibits a relatively low melting point (117 °C), consequently, the resulting oxicam–MTF salts display melting points lower than the oxicams, as observed for **MLX–MTF** and **TNX–MTF**. In contrast, **PRX–MTF** exhibits a melting point higher than both parent components, which represents a less common but still reported behavior.^87^

Although our computational studies indicate that **TNX–MTF** exhibits the most favorable hydrogen-bonding interactions, this salt displays the lowest melting point. This apparent discrepancy highlights the role of additional stabilizing forces. In **PRX–MTF** and **MLX–MTF**, despite having comparatively weaker hydrogen-bond networks, the crystal packing is reinforced by stronger π–π and C–H···π contacts. These interactions are known to significantly influence melting behavior, effectively compensating for the lower hydrogen-bond contributions and resulting in a superior thermal performance compared to **TNX–MTF**.

Of particular note is the enhanced thermal stability of the salts compared to **MTF**, which is known to be thermally labile (**MTF** mp = 117 °C).^12^ Remarkably, **MLX–MTF** even surpassed the thermal stability of **MTF·HCl**, while offering potential dual anti-inflammatory and antihyperglycemic activity.

### 3.6 Thermodynamic Stability

The thermodynamic stability of the new molecular salts was evaluated through aqueous SL experiments conducted under two conditions simulating the gastrointestinal tract: pH 1.2 (mimicking the stomach) and pH 6.8 (near-neutral, mimicking the intestine). After 24 h of equilibration at room temperature, the suspensions were filtered, and the solids were analyzed by PXRD (Figure S6). **PRX–MTF** and **TNX–MTF** remained crystalline and stable under both pH conditions, indicating resistance to dissociation, polymorphic transformation, or hydration. In contrast, **MLX–MTF** exhibited signs of instability at pH 1.2, as evidenced by the appearance of **MLX** in the PXRD pattern, thus indicating the dissociation of the salt. The parent APIs, **TNX** and **MLX**, also maintained their crystallinity and stability. In contrast, **PRX** alone showed noticeable changes in its PXRD pattern both at pH 6.8 and 1.2, consistent with the formation of a hydrated form. Comparison with the PXRD pattern of **PRX** monohydrate from the CSD (CCDC n° 1124947) confirmed this transformation. Interestingly, **PRX–MTF** remained unaffected by hydration under identical conditions. This suggests that **MTF** plays a stabilizing role in the crystal lattice, due to the already described strong intermolecular interactions that involve the hydrophilic functional groups, thus restricting water uptake.

Accelerated aging studies were also conducted to assess solid-state stability under stress conditions (40 °C, 75% RH). After two months of storage, PXRD analysis revealed no significant changes in crystallinity or phase composition for any of the salts or their parent oxicams, confirming excellent physical stability under high humidity and temperature (Figure S7). In contrast, **MTF** liquefied within 4 h under humid conditions (Figure S8a), and after one **MTF** was found to oxidize, developing an orange coloration (Figure S8b). These results highlight the intrinsic reactivity and poor solid-state stability of **MTF**. This molecule requires the presence of a counterion to achieve a stable form, as observed in the commercial salt **MTF·HCl**. In this work, however, we effectively overcome the inherent instability of **MTF** through salt formation with oxicams, generating a drug–drug material with combined pharmacological activity. This stabilization effect is consistent with previous reports in the field of multicomponent solid forms, where salt formation or cocrystallization has been shown to improve the solid-state stability of thermolabile and hygroscopic drugs.^12,23,88,89^

### 3.7 Solubility

The solubility of the compounds was evaluated using a UV/vis spectrophotometer at pH values of 1.2 and 6.8 at room temperature. The focus of these studies was the solubility of the oxicam molecules—known for their limited aqueous solubility—since **MTF** has previously demonstrated excellent solubility in aqueous media. Therefore, the results presented here correspond to the experimental solubility of the native oxicams and the oxicams concentrations obtained from the respective salts with **MTF**. The maximum absorbance peaks used in this analysis were 358 nm for **PRX**, 366 nm for **MLX** and 370 nm for **TNX**.

Figure 12 presents the solubility profiles of the oxicams and their corresponding oxicam–MTF salts in KCl buffer (pH 1.2) and PBS buffer (pH 6.8). Under acidic conditions, a general “spring–parachute” behavior was observed within the first 15 min for all materials, which is usually associated with enhanced absorption.^90–92^ This effect was most pronounced for **PRX–MTF**, which exhibited a maximum solubility of 0.156 mg/mL. Native **PRX** reached a similar peak solubility of 0.135 mg/mL at 3 h; however, due to its instability in acidic media, hydration phenomena led to a reduction in solubility to 0.036 mg/mL. The equilibrium solubility of the **PRX–MTF** salt under these conditions was 0.034 mg/mL, indicating no significant improvement over the native drug, aside from the enhanced stability confirmed previously via PXRD analysis. In the case of **MLX**, the native drug reached a maximum and equilibrium concentration of 0.004 mg/mL. In contrast, the **MLX–MTF** salt reached a peak concentration of 0.030 mg/mL within 30 min, an approximately 10-fold increase. However, as expected, its solubility declined thereafter to 0.002 mg/mL, similar to that of the native **MLX**, due to the low stability of the **MLX–MTF** salt under acidic conditions already observed in the stability section. For **TNX–MTF**, no significant enhancement in solubility was observed. The salt reached a maximum concentration of 0.084 mg/mL initially, stabilizing at 0.070 mg/mL, which is nearly identical to the equilibrium solubility of native **TNX**. These findings are consistent with previously reported solubility values of the native oxicams, lending further credibility to our results: **PRX** (0.019 mg/mL), **MLX** (0.040 mg/mL), and **TNX** (0.031 mg/mL).^93,94^

**Figure 12:**
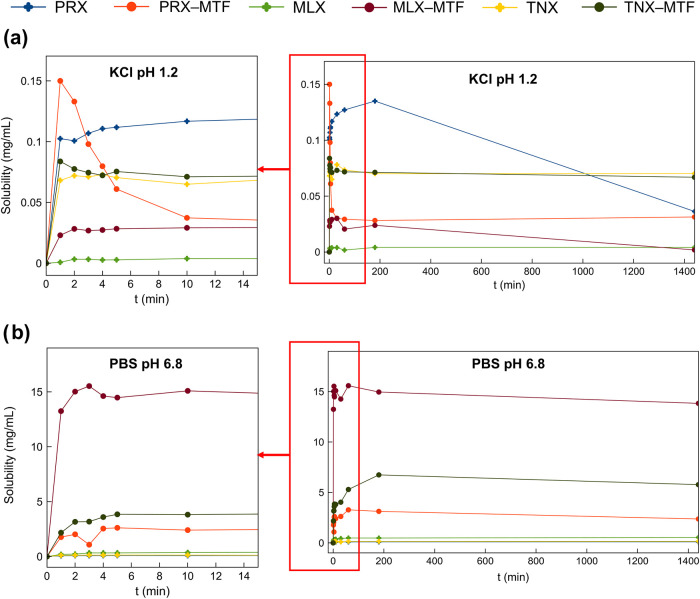
Solubility profiles of **PRX–MTF**, **MLX–MTF**, **TNX–MTF** and their corresponding APIs under (a) pH 1.2 (KCl) and (b) pH 6.8 (PBS) conditions. The graphs on the left in panels (a, b) correspond to the enlarged sections highlighted by the red boxes.

In PBS buffer at pH 6.8, more promising results were obtained, which is particularly relevant as oxicams are primarily absorbed in the intestinal tract. A substantial increase in solubility was observed for all three salts compared to their respective native APIs. **PRX–MTF** reached a maximum solubility of 3.276 mg/mL within the first hour—approximately 33 times greater than native **PRX**. Its equilibrium solubility was 2.370 mg/mL, still about 23 times higher than **PRX** (0.103 mg/mL). **MLX–MTF** presented an over 30-fold increase in solubility when compared to **MLX**, reaching a concentration of 15.529 mg/mL in just 3 min, compared to the maximum solubility of **MLX** at 0.541 mg/mL after 24 h, which also denoted an improvement in the dissolution rate. Similarly, **TNX–MTF** reached a peak solubility of 6.775 mg/mL, representing a 46-fold improvement over native **TNX** (0.154 mg/mL). Interestingly, in PBS, the native APIs did not exhibit a significant “spring–parachute” effect, whereas the salts did. This is consistent with the rapid release of the highly soluble **MTF** component from the salt structure, promoting rapid supersaturation of the oxicam and leading to higher peak concentrations. Interestingly, in their native structures, oxicams molecules form strong intermolecular dimers, which are directly linked to their poor aqueous solubility. The incorporation of highly soluble **MTF** molecules disrupts these oxicam**–**oxicam dimers, providing a rational explanation for the improved solubility and dissolution profile observed for the oxicam**–MTF** salts.

To contextualize the remarkable solubility enhancement achieved with **MTF**, our results were compared with previously reported oxicam salts and cocrystals. For example, Huang et al. reported pharmaceutical salts of **PRX** and **MLX** with 4-aminopyridine, 4-dimethylaminopyridine, and piperazine, which showed moderate solubility increases for **PRX** (≈2.8-fold for PRX–4AP and PRX–4DMP) and higher, yet still limited, enhancements for **MLX** (≈3–10-fold, depending on the counterion) under PBS conditions.^43^ Other **MLX** salts with amino acids have been reported to yield solubility improvements of approximately 14-fold in acidic media.^95^ For **TNX**, Bolla et al. described a TNX–resorcinol cocrystal exhibiting a 10-fold increase in solubility, while a TNX–piperazine salt showed a 5.5-fold enhancement relative to the parent API under comparable conditions.^93^

Overall, these reported results do not allow a clear distinction as to whether salt or cocrystal formation alone is responsible for major solubility improvements. Moreover, the acidic or basic nature of the coformer does not solely determine solubility enhancement, as this property is primarily governed by the crystal structure of the resulting multicomponent material. Consequently, detailed structural analyses and computational studies are required to rationalize solubility behavior and to elucidate the role of the coformer.

Although the primary aim of this study was to improve oxicam solubility, the stability of the salts also provides insight into solubility modulation when compared with **MTF**. In PBS, the solubility of the **PRX–MTF**, **MLX–MTF**, and **TNX–MTF** salts was reduced by more than 300, 50, and 125-fold, respectively, compared to **MTF·HCl** (256–269 mg/mL).^96^ This modulation in **MTF** is a direct consequence of the strong hydrogen bonds established with the oxicams, which reduced direct water interaction and delayed rapid dissolution. This downregulation can also offer therapeutic advantages, as one of the major clinical issues associated with **MTF** is gastrointestinal irritation due to its high solubility and local accumulation.

## 4 Conclusions

In this work, we have successfully obtained and characterized three novels drug–drug pharmaceutical salts of **PRX**, **MLX**, and **TNX**, using **MTF** as coformer. These salts allowed to improve the solubility of the oxicams at physiological pH (reaching up to 46-fold increases for **TNX–MTF** at pH 6.8), while simultaneously moderating the excessive solubility of **MTF**. Additionally, an improved dissolution behavior was observed for **PRX–MTF** and **MLX–MTF**, characterized by a spring–parachute profile. This dual modulation may enhance oxicam bioavailability while reducing **MTF**-associated gastrointestinal side effects. Beyond solubility, the salts demonstrated excellent stability. Thermal and humidity stress tests confirmed superior solid-state stability of the salts compared with **MTF**. Moreover, **PRX–MTF** also showed resistance to hydration, a notable advantage over **PRX**, known for its tendency to hydrate. These findings provide a strong basis for future investigations into the therapeutic and synergistic potential of these drug–drug salts.

Structural determination of the salts provided a rational explanation of the observed pharmacokinetic features. SCXRD and computational analyses confirmed that salt formation is stabilized through N–H···O, N–H···N, and complementary C–H···π interactions. While **PRX–MTF** and **MLX–MTF** display closely related packing motifs with comparable balances of hydrogen bonding and π-interactions, **TNX–MTF** is distinguished by a stronger hydrogen-bonding network but reduced π-stabilization. Importantly, **MTF** intercalates between oxicam molecules, disrupting the oxicam–oxicam dimers characteristic of their native structures. This disruption is a key factor for the improved solubility and dissolution profiles of the oxicams. At the same time, the oxicam framework shields **MTF** from environmental exposure, enhancing its stability.

Finally, the distinctive fluorescence profiles of the salts were characterized, demonstrating how variations in crystal packing and orbital localization tune their optical behavior. The 33 nm red-shift in **PRX–MTF** is supported by HOMO localization on the enolic group, which is directly involved in the hydrogen-bonding network and π-stacking. In contrast, the complete fluorescence quenching observed in **MLX–MTF** is attributed to the nearly perpendicular orientation of the **MTF** ^**+**^ cation, which promotes nonradiative deactivation. For **TNX–MTF**, the shift in orbital localization to the five-membered ring and the lower calculated energy gap of 2.364 eV provide a quantitative basis for its broadened green emission. Overall, this work aims to demonstrate that deliberate salt formation between **MTF** and oxicam provides a powerful strategy to overcome key challenges, combining enhanced solubility, stability, and potential therapeutic synergy effects. By directly linking crystal structure features with pharmaceutical performance, this study underscores the value of crystal engineering in the development of PMMs with dual therapeutic effects.

## Supplementary Material



## Notes

Table and figures comprehensively document: (1) TGA and DSC profiles of PRX–MTF, MLX–MTF and TNX–MTF; (2) PXRD patterns of PRX, MLX, TNX and their respective salts with MTF after stirring in PBS pH 6.8 and KCl pH 1.2 solutions for 24 h at 25 °C; (3) PXRD patterns of PRX, MLX, TNX and their respective salts with MTF under accelerated aging conditions at different time intervals; (4) images of MTF under accelerated aging conditions at different time intervals (PDF)
