# Supplementary material for: Multicomponent Oxicam–Metformin Salts: Toward a Strategy for Enhancing Solubility and Stability
Source: Cryst Growth Des. 2026 Feb 21;26(5):1989–2004. doi: 10.1021/acs.cgd.5c01574 (PMC12965098; doi:10.1021/acs.cgd.5c01574)
Supplement: Supplementary file 1 [file cg5c01574_si_001.pdf]

Multicomponent Oxicam–Metformin Salts: Towards a Strategy for Enhancing Solubility and Stability.

Estephany Muñoz-Hernández[1,2], Carolina Alarcón-Payer[3], Antonio Frontera[4], Antonio Rodríguez-Diéguez[5], Francisco J. Acebedo-Martínez[1,] Alicia Domínguez-Martín[2], Duane Choquesillo-Lazarte[1],**

[1] Laboratorio de Estudios Cristalográficos, IACT-CSIC, Avda. de las Palmeras 4, 18100 Armilla, Spain.

[2] Department of Inorganic Chemistry, Faculty of Pharmacy, University of Granada, 18071 Granada, Spain.

[3] Servicio de Farmacia, Hospital Universitario Virgen de las Nieves, 18014 Granada, Spain.

[4] Departament de Química, Universitat de les Illes Balears, Crta. de Valldemossa km 7.5, 07122 Palma, Spain.

[5] Department of Inorganic Chemistry, Faculty of Sciences, University of Granada, Granada 18071, Spain.

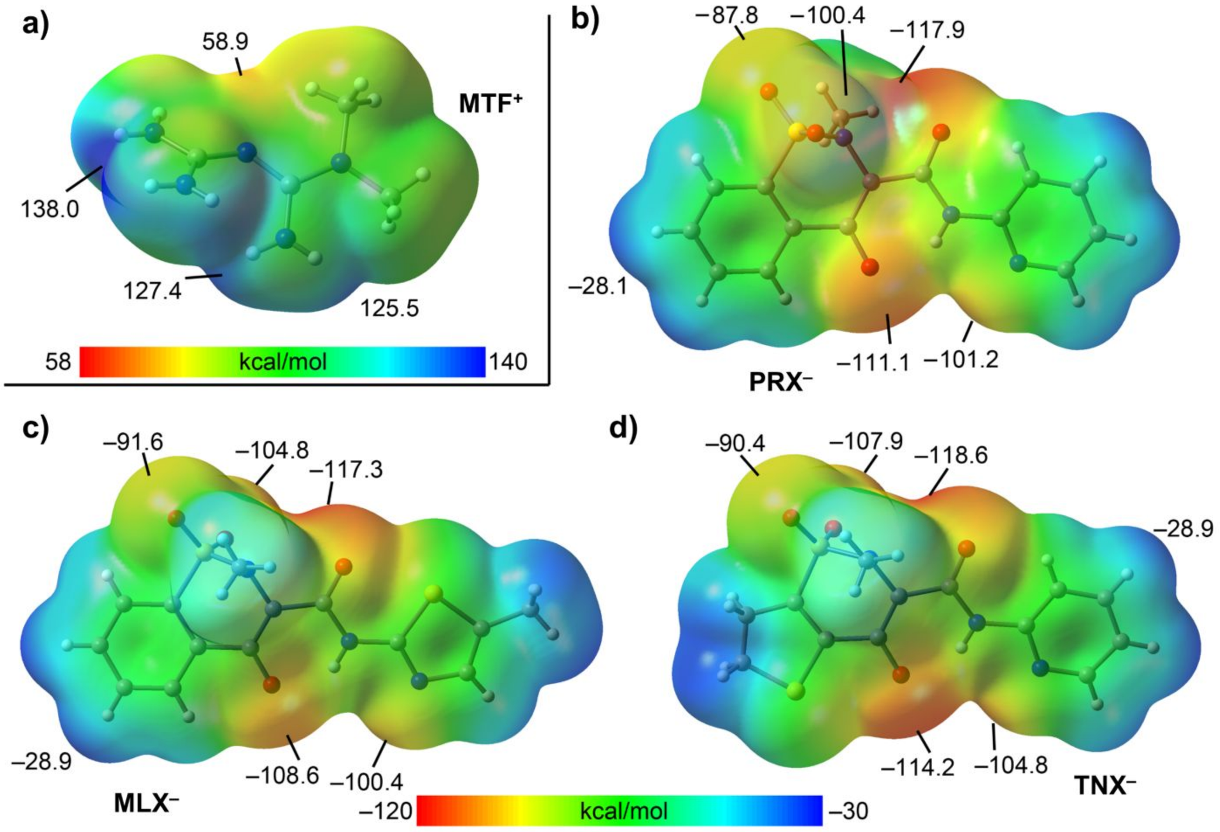

Figure S1. MEP surfaces for a) **MTF⁺**, b) **PRX⁻**, c) **MLX⁻**, and d) **TNX⁻**. The colour scale ranges from red, most negative potential to blue, most positive potential. Key MEP values (in kcal/mol) are labelled on the surfaces. Isovalue 0.001 a.u.

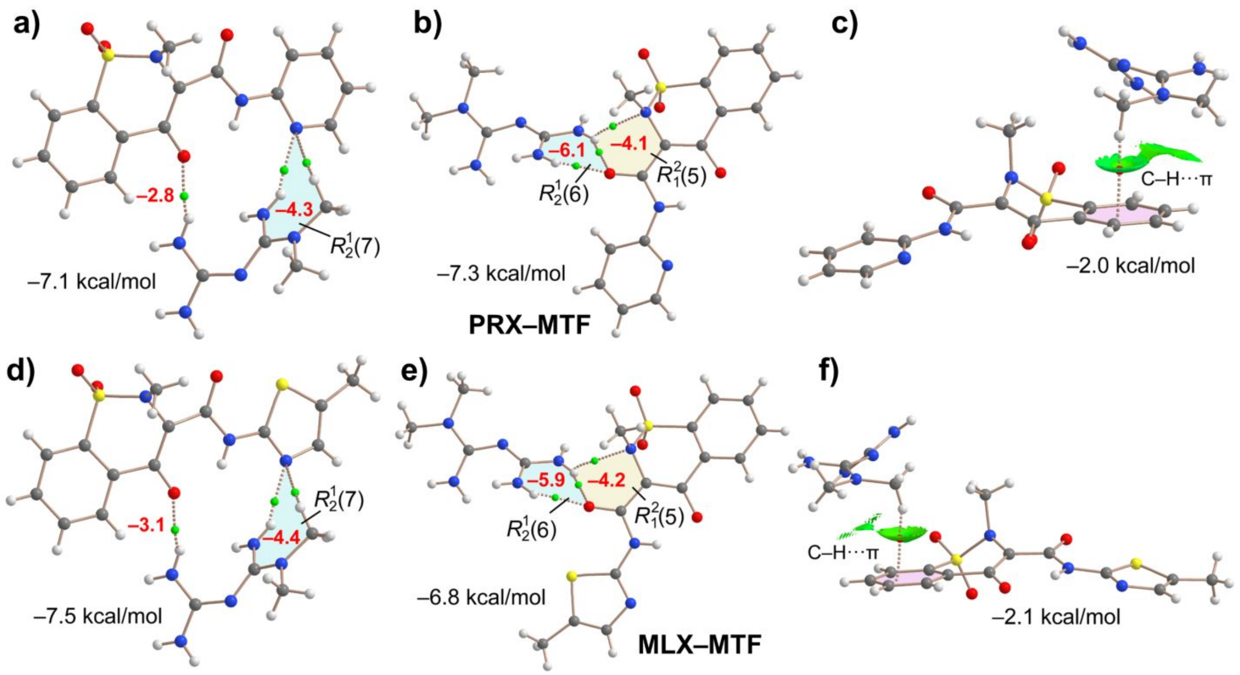

Figure S2. QTAIM analysis and selected NCIplot visualization of dimeric assemblies extracted from SCXRD structures of a,b,c) **PRX–MTF** and d,e,f) **MLX–MTF** salts. Green spheres are H-bond BCPs, red spheres are C–H···π BCPs. Interaction energies are indicated.

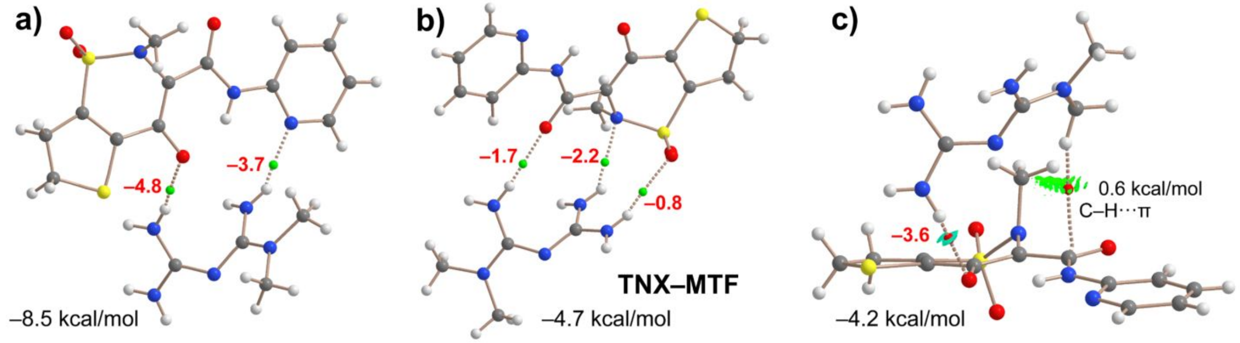

Figure S3. a,b) QTAIM analysis and c) QTAIM/NCIplot visualization of dimeric assemblies extracted from the X-ray structure of **TNX–MTF**. Interaction energies are indicated.

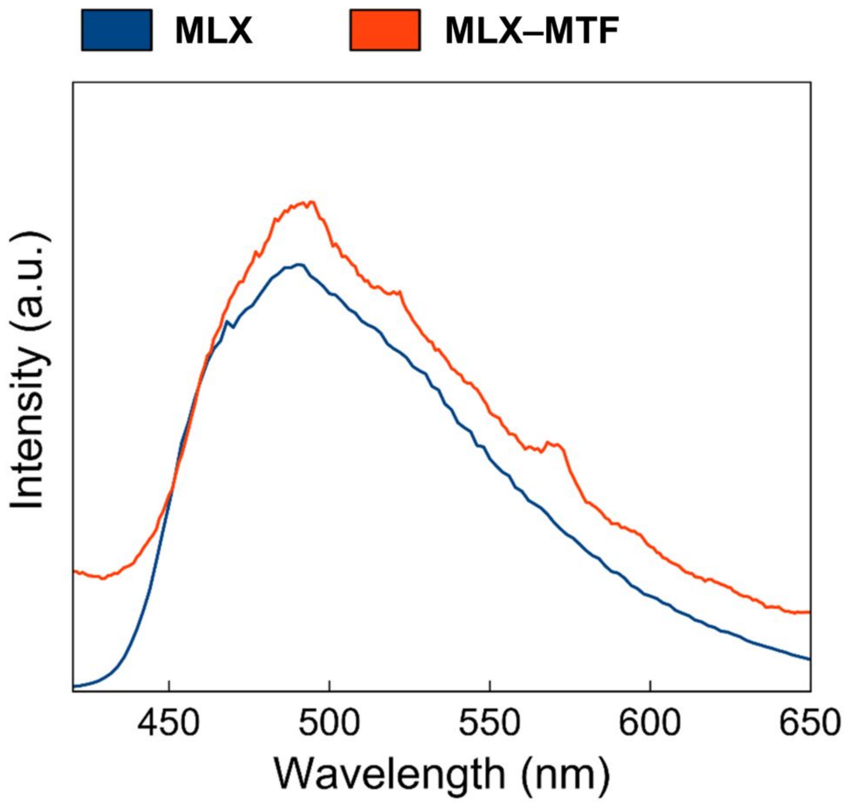

Figure S4. Solid-state fluorescence emission spectra of **MLX–MTF** using 5/5 nm slits.

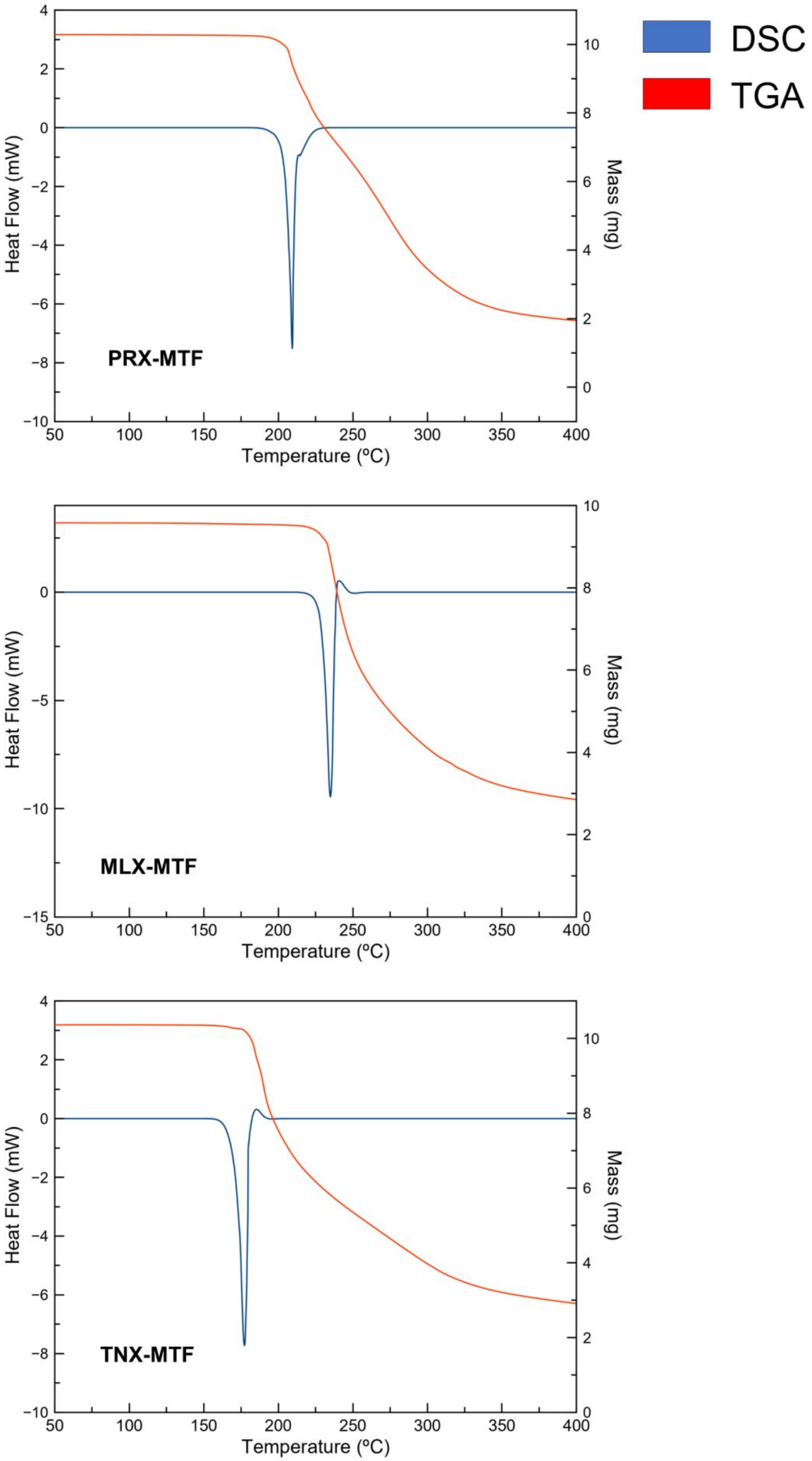

Figure S5. TGA and DSC profiles of PRX–MTF, MLX–MTF and TNX–MTF.

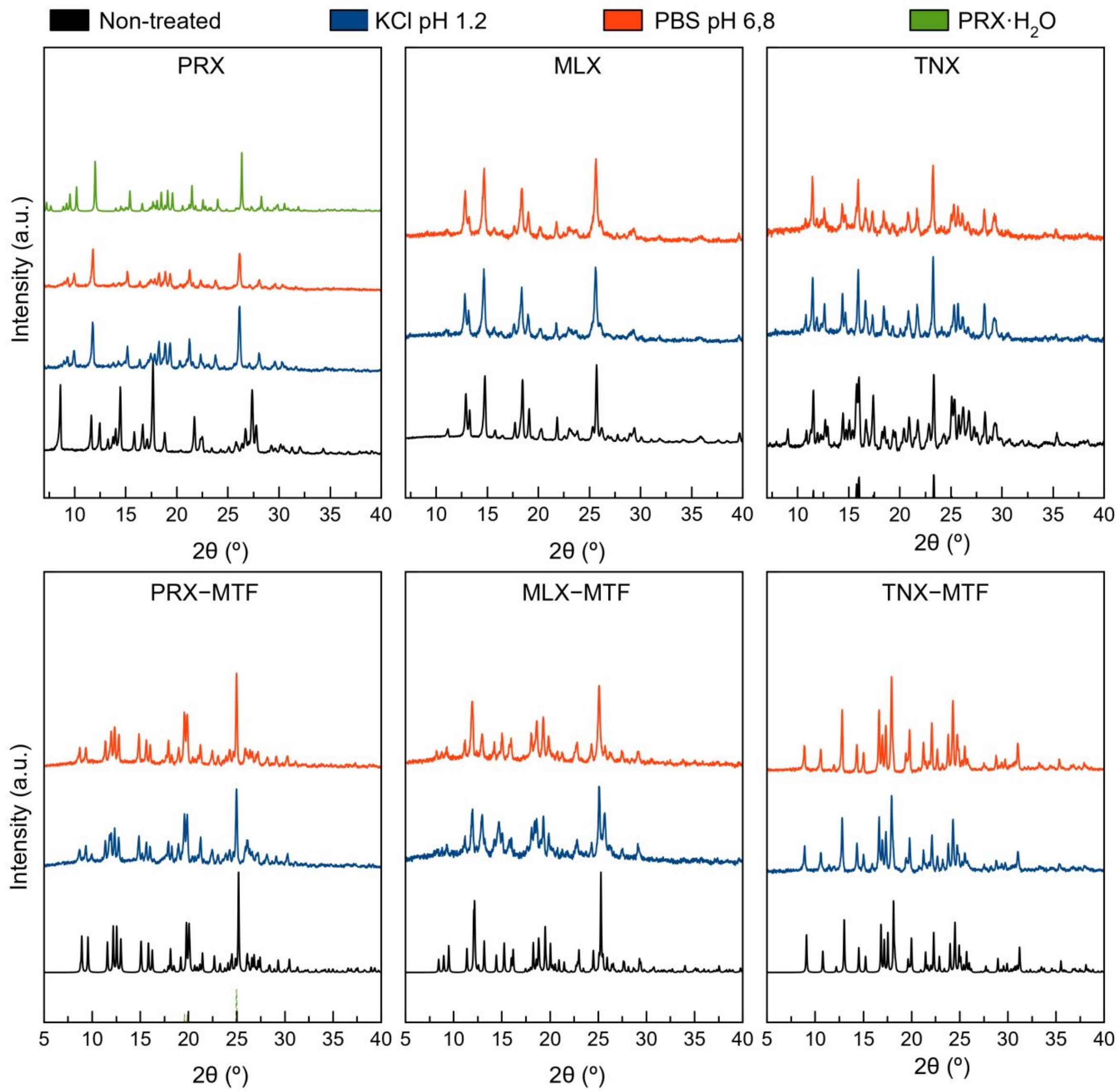

Figure S6. PXRD patterns of **PRX**, **MLX**, **TNX** and their respective salts with **MTF** after stirring in PBS pH 6.8 and KCl pH 1.2 solutions for 24 hours at 25 °C.

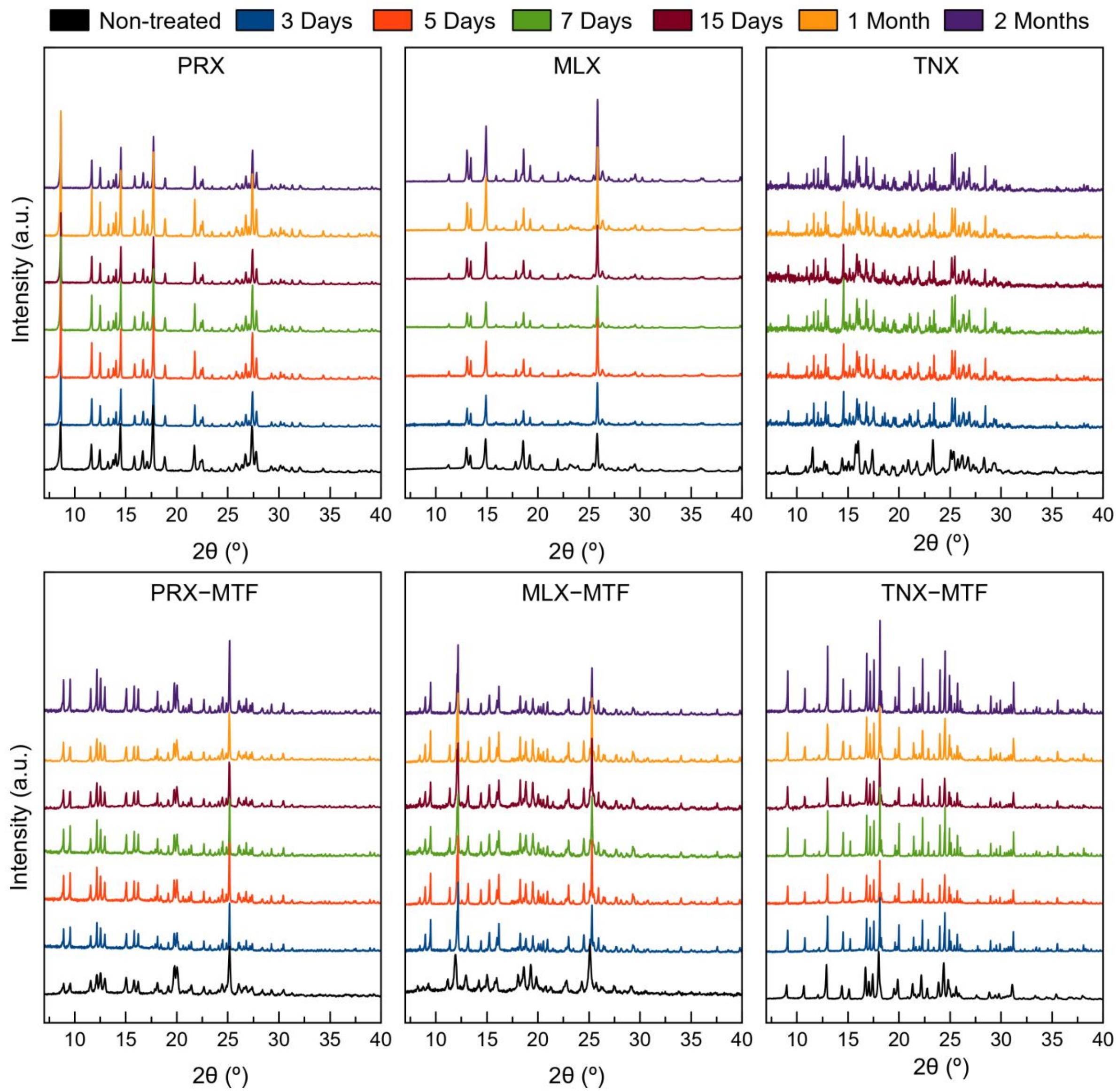

Figure S7. PXRD patterns of **PRX**, **MLX**, **TNX** and their respective salts with **MTF** under accelerated aging conditions (40 °C, 75 %RH) at different time intervals.

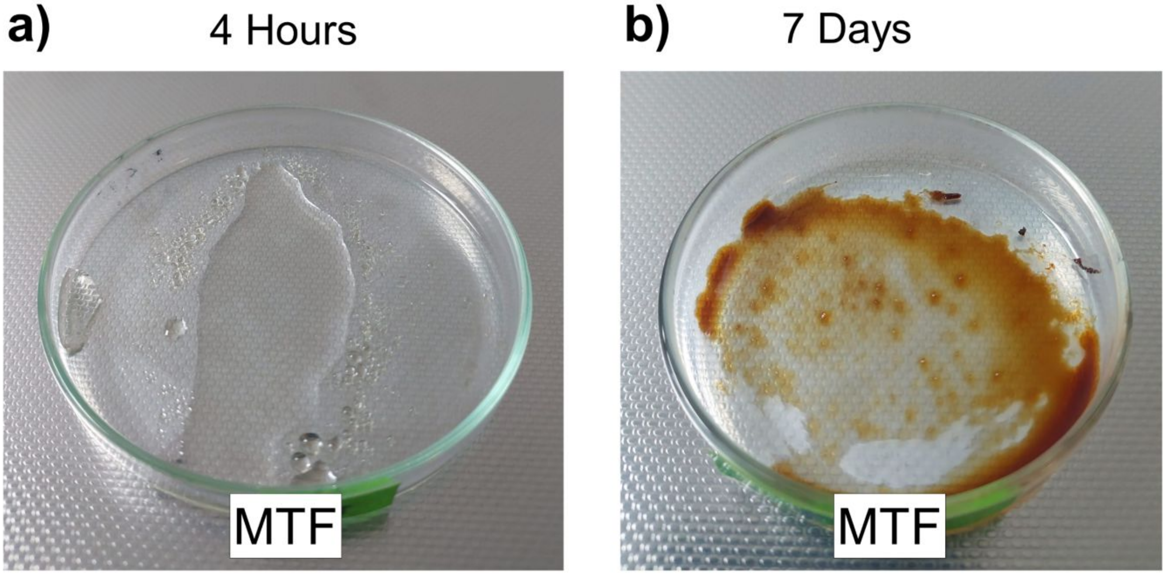

Figure S8. Images of **MTF** under accelerated aging conditions (40 °C, 75 %RH) at different time intervals.
